# Genetic Inhibition of Phosphorylation of the Translation Initiation Factor eIF2α Does Not Block Aβ-Dependent Elevation of BACE1 and APP Levels or Reduce Amyloid Pathology in a Mouse Model of Alzheimer’s Disease

**DOI:** 10.1371/journal.pone.0101643

**Published:** 2014-07-03

**Authors:** Katherine R. Sadleir, William A. Eimer, Randal J. Kaufman, Pavel Osten, Robert Vassar

**Affiliations:** 1 Department of Cell and Molecular Biology, Northwestern University, Feinberg School of Medicine, Chicago, Illinois, United States of America; 2 Department of Neurology, Massachusetts General Hospital, Charlestown, Massachusetts, United States of America; 3 Sanford|Burnham Medical Research Institute, La Jolla, California, United States of America; 4 Cold Spring Harbor Laboratory, Cold Spring Harbor, New York, United States of America; University of S. Florida College of Medicine, United States of America

## Abstract

β-site amyloid precursor protein (APP) cleaving enzyme 1 (BACE1) initiates the production of β-amyloid (Aβ), the major constituent of amyloid plaques in Alzheimer’s disease (AD). BACE1 is elevated ∼2–3 fold in AD brain and is concentrated in dystrophic neurites near plaques, suggesting BACE1 elevation is Aβ−dependent. Previously, we showed that phosphorylation of the translation initiation factor eIF2α de-represses translation of BACE1 mRNA following stress such as energy deprivation. We hypothesized that stress induced by Aβ might increase BACE1 levels by the same translational mechanism involving eIF2α phosphorylation. To test this hypothesis, we used three different genetic strategies to determine the effects of reducing eIF2α phosphorylation on Aβ-dependent BACE1 elevation in vitro and in vivo: 1) a two-vector adeno-associated virus (AAV) system to express constitutively active GADD34, the regulatory subunit of PP1c eIF2α phosphatase; 2) a non-phosphorylatable eIF2α S51A knockin mutation; 3) a BACE1-YFP transgene lacking the BACE1 mRNA 5′ untranslated region (UTR) required for eIF2α translational regulation. The first two strategies were used in primary neurons and 5XFAD transgenic mice, while the third strategy was employed only in 5XFAD mice. Despite very effective reduction of eIF2α phosphorylation in both primary neurons and 5XFAD brains, or elimination of eIF2α-mediated regulation of BACE1-YFP mRNA translation in 5XFAD brains, Aβ-dependent BACE1 elevation was not decreased. Additionally, robust inhibition of eIF2α phosphorylation did not block Aβ-dependent APP elevation in primary neurons, nor did it reduce amyloid pathology in 5XFAD mice. We conclude that amyloid-associated BACE1 elevation is not caused by translational de-repression via eIF2α phosphorylation, but instead appears to involve a post-translational mechanism. These definitive genetic results exclude a role for eIF2α phosphorylation in Aβ-dependent BACE1 and APP elevation. We suggest a vicious pathogenic cycle wherein Aβ42 toxicity induces peri-plaque BACE1 and APP accumulation in dystrophic neurites leading to exacerbated Aβ production and plaque progression.

## Introduction

The amyloid plaque, a hallmark of Alzheimer’s disease (AD) pathology, is composed of a core of fibrillar 38–43 amino acid β-amyloid Aβ peptides [1], [2]). Soluble oligomeric forms of Aβ are also thought to be present at high levels in the AD brain [3]. A vast amount of evidence suggests that 42-amino acid Aβ (Aβ42) has a crucial early role in the pathogenesis of AD [1], [2]. Oligomeric Aβ42 has many toxic effects such as inhibition of long-term potentiation in the hippocampus [4], [5], alteration of synaptic structure and synaptic transmission [6]–[11], and cytoskeletal damage [12], among other effects in vitro and in vivo. Aβ peptides are generated by the sequential proteolytic processing of the transmembrane protein, Amyloid Precursor Protein (APP) by the β-secretase enzyme β-site APP Cleaving Enzyme 1 (BACE1), which cuts APP first, and the γ-secretase complex, which contains the intramembrane aspartic protease presenilin (PS1, PS2) [13].

There is considerable genetic evidence suggesting that Aβ42 is the causative agent in Alzheimer’s disease. Mutations in APP, PS1 and its homolog, PS2, cause an early onset autosomal dominant form of Familial Alzheimer’s Disease (FAD) [2]. Over 200 mutations in the genes for APP, PS1, and PS2 have been identified that increase production of total Aβ or Aβ42, or increase Aβ aggregation, and cause FAD. FAD can also be caused by specific duplication of the APP gene locus [14], [15], or by Trisomy 21/Downs syndrome [2]. The FAD mutations increase the rate of secretase cleavage of APP resulting in greater Aβ production [16]. For example, the Swedish mutation (K670N, M671L) at the BACE1 cleavage site in APP makes APP a better BACE1 substrate, and thus increases BACE1 cleavage and raises total Aβ generation to cause FAD [17]. This implies that increased BACE1 activity might result in AD. Moreover, except for age of onset, the phenotypes of FAD and sporadic AD are highly similar, strongly suggesting that Aβ has a critical early role in the more common sporadic form of AD. Recently, a mutation in APP near the BACE1 cleavage site (A673T) was identified that protects against AD by reducing BACE1 cleavage of APP and Aβ production [18]. These results taken together suggest that therapeutic reduction of the activities of the secretases, especially BACE1, should lower Aβ levels, which might be beneficial for AD.

It is consistently observed that levels of BACE1 protein and activity are elevated in the brains of AD patients [19]–[23] and APP transgenic (Tg) mice [22], [24]–[26], suggesting that increased BACE1 could have a role in the development and/or progression of AD by exacerbating Aβ generation. While the mechanism of BACE1 elevation in AD and APP Tg brains is not fully understood, recent data indicate that BACE1 levels are upregulated during stresses associated with AD risk such as energy deprivation [22], [27], hypoxia and stroke [28]–[30], oxidative stress [31] and traumatic brain injury [32], [33]. Several molecular pathways have been proposed to mediate the BACE1 elevation, such as caspase-3-induced cleavage of the trafficking adaptor protein GGA3 leading to impaired lysosomal degradation of BACE1 [34], [35], Cdk5 phosphorylation of transcription factor Stat3 [36], altered levels of microRNAs [37]–[41], increased transcription factor HIF1α activity [29], and elevated BACE1 mRNA translation via phosphorylation of the translation initiation factor eIF2α [22].

In the brains of APP Tg mice and AD patients, high concentrations of BACE1 protein accumulate in swollen dystrophic neurites in very close proximity to amyloid plaques [22], [24], [26], suggesting that Aβ itself is responsible for raising BACE1 levels [42]. However, the complexity of the regulation of BACE1 levels in neurons has made it difficult to discern the molecular and cellular mechanism(s) involved in Aβ-dependent BACE1 elevation. Our previous work using primary neuron cultures indicates that Aβ42 oligomers directly elevate BACE1 levels through a post-transcriptional mechanism independent of caspase-3 and Cdk5 activity [25]. Since these molecules did not seem to play a key role in Aβ42-induced BACE1 elevation, we focused on the eIF2α phosphorylation pathway, which we have previously shown increases BACE1 levels via enhanced translation of BACE1 mRNA following energy inhibition [22]. In primary neurons, compounds that increase eIF2α phosphorylation, such as the PP1c phosphatase inhibitor salubrinal, also increase BACE1 levels and Aβ generation. In BACE1 overexpressing HEK cells, genetic reduction of eIF2α phosphorylation prevents the BACE1 increase associated with energy inhibition, further verifying this mechanism. The positive correlation of levels of BACE1, phosphorylated eIF2α, and amyloid load in human brain along with the significant increase of BACE1 and phospho-eIF2α levels in AD brains compared to non-demented controls [22] suggests the eIF2α phosphorylation pathway might be relevant to AD pathogenesis. Lastly, the increase of both BACE1 and phospho-eIF2α levels in the 5XFAD transgenic mouse model of aggressive amyloid pathology [22] indicates that this animal model could be useful for studying the role of eIF2α phosphorylation in BACE1 elevation and amyloidogenesis in AD.

eIF2α is phosphorylated in response to certain cell stressors such as viral infection, hypoxia, low nutrients and accumulation of unfolded proteins in the ER. Phosphorylation of eIF2α reduces global translation of transcripts, but increases translation of specific stress-response mRNAs that encode proteins that limit damage and assist the cell during stress recovery. The eIF2 complex functions by binding GTP and initiator tRNA, then scanning the mRNA until it reaches a start codon where it hydrolyzes GTP and dissociates while the ribosome continues translation. Normally, eIF2B is the guanine nucleotide exchange factor for eIF2, but when the α subunit of eIF2 is phosphorylated at serine 51 (S51), eIF2 binds tightly to eIF2B thus inhibiting its guanine nucleotide exchange activity and eIF2 is unable to load GTP and initiate translation. As a result of eIF2α phosphorylation, certain mRNA transcripts with long 5′ untranslated regions (UTRs) that contain short upstream open reading frames (uORFs) are translated with increased efficiency because ribosomes have an increased probability of scanning through the uORFs to re-initiate translation at the true start codon [43], [44]. Similarly, the BACE1 transcript has a long, GC-rich 5′ UTR that contains three uORFs that represses translational efficiency under basal conditions [45]–[48]. eIF2α phosphorylation that is induced by energy deprivation causes de-repression of BACE1 mRNA translation, thus resulting in increased BACE1 protein levels [22].

Here, we employed three different genetic strategies to determine the effects of reduced eIF2α phosphorylation on Aβ-dependent BACE1 elevation in vitro and in vivo: 1) a two-vector adeno-associated virus (AAV) system to express constitutively active GADD34, a regulatory subunit of the PP1c eIF2α phosphatase; 2) a non-phosphorylatable eIF2α S51A knockin mutation [49]; 3) a BACE1-YFP transgene lacking the BACE1 mRNA 5′ UTR that is required for translational regulation by phosphorylated eIF2α. The first two strategies were used in primary neuron cultures and 5XFAD transgenic mouse brains, while the third strategy was employed only in 5XFAD transgenic mice. We report that despite very effective reduction of phosphorylated eIF2α levels in both primary neuron cultures and 5XFAD brains, or elimination of phosphorylated eIF2α mediated regulation of BACE1-YFP mRNA translation in 5XFAD brains, Aβ-dependent BACE1 elevation could not be decreased. We conclude that the amyloid-associated increase in BACE1 level is not caused by translational de-repression via eIF2α phosphorylation, but instead appears to involve a post-translational mechanism.

## Materials and Methods

### AAV constructs

GADD34 constitutive active N-terminal truncation and GADD34 control C-terminal truncation constructs were a generous gift of Dr. David Ron. GADD34 CA and GADD34 control cDNAs were expressed from a recombinant adeno-associated virus (AAV)-based two-vector system. Each cDNA was cloned into an AAV “responder vector” consisting of a tTA-regulated tet-operator-CMV (tetO-CMV) bidirectional promoter driving coexpression of GADD34 CA and eGFP or GADD34 control and eGFP. Expression from the responder vector is activated only in the presence to the tetracycline transactivator protein (tTA), which is expressed from an “activator vector” under the control of the forebrain pyramidal neuron-specific calmodulin kinase II (CaMKII) promoter [50].

### Mice

5XFAD mice were generated as previously described [51]. The 5XFAD line was maintained by crossing transgene positive males with B6/SJL F1 hybrid females from Jackson Laboratories. The eIF2α S51A mice were generated as described [49], and maintained by crossing S/A heterozygous mice to wild type mice. BACE1-YFP were generated as described [52]. Briefly, eYFP was fused to the coding region of human BACE1, and cloned into the PMM400 tetO expression vector (gift of M. Mayford, The Scripps Institute, La Jolla, CA). This construct was used to make tetO promoter BACE1-YFP transgenic mouse lines, which were then crossed to transgenic mice expressing the tet transactivator (tTA) from the forebrain pyramidal neuron-specific CaMKII promoter (gift of M. Mayford) generating CaMKII:BACE1-YFP bigenic mice that express BACE1-YFP in forebrain neurons (referred to as BACE1-YFP mice). These bigenic females were crossed to 5XFAD males to generate 5XFAD;BACE1-YFP and non-5XFAD BACE1-YFP littermates. For all experiments, cohorts consisted of approximately 50% male and 50% female mice for each genotype, except for all BACE1-YFP and 5XFAD;BACE1-YFP mice were female. In no experiment did we observe a difference in BACE1 level or phospho:total eIF2α ratios between the sexes, so analyses of males and females were combined. All mice were genotyped by PCR amplification of tail clip DNA. Mice were sacrificed by carbon dioxide inhalation or by a lethal dose of ketamine/xylazine, followed by perfusion with ice cold 1xPBS containing protease and phosphatase inhibitors. One hemibrain was either snap-frozen whole in liquid nitrogen, or dissected into hippocampus and cortex which were snap-frozen separately. Snap frozen hemibrains, cortices or hippocampi were homogenized in 1xPBS with 1% Triton X-100 supplemented with protease inhbitors (Calbiochem) and Halt Phosphatase Inhibitor Cocktail (Thermo Scientific). Protein concentration was quantified using BCA Assay (Pierce). The other hemibrain was drop fixed in 4% paraformaldehyde in PBS for 16–20 hrs, then transferred to 20% w/v sucrose in 1xPBS for 24 hours, then stored in 30% w/v sucrose in 1xPBS with azide. All animal work was done with the approval of the Northwestern University IACUC, assurance number A3283-01.

### AAV injection

Serotype 1 AAV was prepared by VectorBioLabs (Philadelphia, PA). Genomic titer was determined by quantitative PCR. 5XFAD transgene positive males were crossed to SJL/B6 hybrid females in timed matings to generate transgene negative and positive littermates. On P0, each pup in a litter was cryoanesthatized and injected with 2 µl containing 6.6×10^10^ viral genomes of GADD34 control-AAV or GADD34 CA-AAV and 6.9×10^10^ viral genomes of CamKII-tTA activator vector into each hemisphere, using a 10 µl Hamilton syringe, as described in [53]. Pups were returned to the mother and aged to 1, 3, or 6 months.

### Primary neurons, AAV and Aβ42

Timed matings between C57/B6 pairs or eIF2α S/A heterozygous pairs were set up for two nights. Cortical neurons were isolated from day 15.5–16.5 mouse embryos via dissociation at 37°C in 0.25% trypsin. For immunoblotting, brains were plated at the density of 750,000 cells per well in 12 well plates previously coated with 1 mg/ml poly-L-lysine (Sigma) in borate buffer. Neurons were plated in neurobasal media supplemented with 2% B-27, 500 µM glutamine, 10% horse serum and 2.5 µM glutamate. After 2–3 hours, this was replaced with growth media (neurobasal media with 2% B-27 and 500 µM glutamine). All cell culture reagents were from Invitrogen. For AAV infection, 1×10^7^ activator viral genomes and 1×10^7^ responder GADD34 control or GADD34 CA viral genomes were added to each well when the plating media was removed and replaced with growth media. AAV for primary neuron experiments was prepared by polyethylenimine transfections of HEK cells with activator or responder GADD34 vectors, and helper plasmids containing the genes required for viral packaging. The helper plasmids used (pDP1, pDP2) [54] generated combined serotype of 1 and 2, which efficiently transduces neurons, and can be purified on a heparin column [55]. Briefly, HEK293T/17 cells (ATCC) were plated in T225 flasks and transfected by linear polyethylenimine (PEI) [56], [57] with equal molar amount of either responder GADD34 cont, GADD34 CA or activator, and pDP1 and pDP2. The cells were collected 72 hours after transfection by scraping. Cells were lysed with freeze/thaw cycles followed by Benzonase and pelleted. The virus containing supernatant was filtered on 0.45 µM Acrodisc syringe filter and loaded onto a heparin column (GE Healthcare). Virus was washed with 1 mM MgCl and 2.5 mM KCl in 1xPBS (PBS-MK) and eluted in PBS-MK with 0.5 M NaCl. The eluted virus was concentrated in Amicon Ultra cut off column (Millipore), washed thoroughly with PBS-MK three times then eluted in 200 µl PBS. Virus titer (genomic particles per ml) was determined by quantitative real-time PCR using RT2 Real-Time SYBR Green/Fluorescein PCR master mix, according to the manufacturer (SuperArray Bioscience).

After 6–8 days in vitro following AAV infection, neurons were exposed to 10 µM oligomeric Aβ42 for 24 or 30 hours or 1 µM Aβ42 for 5 days. Aβ42 oligomers were prepared as described in [58]. Briefly, lyophilized recombinant Aβ42 peptide (rPeptide) was HFIP treated, dried down, then resuspended to 5 mM in dry DMSO and brought to 100 µM in cold, 4 mM HEPES pH 8, incubated on ice at 4°C for 24 hours to generate oligomers [59]. For control cultures, DMSO alone was added to 4 mM HEPES pH 8 and incubated as described. All treatment conditions were done in triplicate. Neurons were lysed on ice in RIPA buffer (150 mM NaCl, 1% IGEPAL CA-630, 0.5% sodium deoxycholate, 0.1% SDS, 50 mM Tris pH 8, 1 mM PMSF) with protease inhibitors (Calbiochem) and Halt Phosphatase Inhibitor Cocktail (Thermo Scientific), spun down 10 minutes at 10,000 RPM at 4°C, and protein in the supernatant quantified by BCA assay (Pierce).

### Immunoblotting

15 or 20 µg of brain homogenate, or 5–10 µg primary neuron lysate were separated on Invitrogen’s 4–12% Bis-Tris NuPage Mini Gels. Protein was transferred to 0.45 µm PVDF membrane, and after transfer stained with 0.1% Ponceau in 5% w/v acetic acid, and scanned. The membrane was rinsed and probed with anti-BACE1 antibody (3D5; 1∶1000) [26], anti-phospho-eIF2α (Epitomics, clone E90, 1∶2000 or Cell signaling #3597, 1∶2000), anti-eIF2α (Cell Signaling #9722, 1∶2000), anti-APP/Aβ (clone 6E10, Covance, 1∶5000, for human APP/Aβ, or clone 22C11, Millipore, 1∶2000, for mouse APP), anti-GFP (Clontech, Living Colors #632375), anti-βIII-tubulin (TuJ1, gift of Dr. Lester Binder, 1∶10,000) anti-β-actin (Sigma clone AC-15, #A5441-1∶30,000), anti-caspase 3 (Cell Signaling #9662-1∶1000), followed by washing and 1 hour incubation with secondary HRP-conjugated anti-mouse or anti-rabbit secondary antibody (Jackson Immunologicals, Vector Laboratories 1∶10,000). Blots were visualized using Amersham’s ECL+ chemiluminescent substrate, or Luminata Crescendo (Millipore), and signals were quantified using a Kodak Image Station 4000 R. Signals were normalized to tubulin or actin signal, or ponceau staining intensity as indicated in figure legends. For analysis of the mouse brain homogenates, gels were cut into horizontal strips and stacked so all samples for a given protein were transferred onto a single piece of PVDF membrane. Putting all samples (up to 106) on one membrane eliminated the need to account for variation in transfer, antibody incubation and ECL application that can occur between blots. For neurons, triplicates of each culture condition were averaged and comparison to control was done using student’s two-tailed t-test using InStat software (GraphPad Software, Inc., San Diego, CA).

### Aβ42 ELISA

Human Aβ42 levels in the 5XFAD AAV-injected cortices were measured using the WAKO Human βAmyloid (1–42) Kit as follows. 7.5 mg/ml brain homogenates were extracted in 5 M guanidine hydrochloride overnight on a rocker, then diluted 1∶50 in PBS with protease inhibitors. All samples were then diluted 1∶100 into Standard Diluent and ELISA was performed according to manufacturer’s instructions.

### Immunofluorescence and microscopy

Paraformaldehyde fixed brains stored in 30% sucrose in PBS were cut into 30 µm coronal or saggital sections on a freezing sliding microtome and collected in PBS. Sections were stained with Thiazine Red (2 mg/ml diluted 1∶60,000) or Thioflavin S (1 mg/ml diluted 1∶5000) for amyloid fibrils, BACE1 (D10E5, Cell Signaling, 1∶500 or Eptomics EPR3956 1∶250), GADD34 (Protein Tech Group, Chicago 1∶1000). GFP and YFP fluorescence signals were imaged directly. Donkey anti-rabbit or anti-mouse conjugated to Alexa 568 or 647 secondary antibody (Molecular Probes) was used at the same concentrations as primary being detected. DAPI (Molecular Probes) was used at 300 nM for imaging nuclei. Coverslips were mounted using Prolong Gold (Molecular Probes), Conventional images were collected using a Keyence BZ9000 microscope (Tokyo, Japan), using 10x or 20x objectives. The “merge” function was used to generate hemibrain and hippocampus images. Confocal images were collected on Nikon A1 microscope using a 60x oil immersion objective lens and NIS Elements software (Northwestern University’s Cell Imaging Facility).

## Results

### Overexpression of constitutively active GADD34 blocks eIF2α phosphorylation but does not prevent Aβ42-induced BACE1 elevation in cultured mouse cortical primary neurons

**Figure 1:**
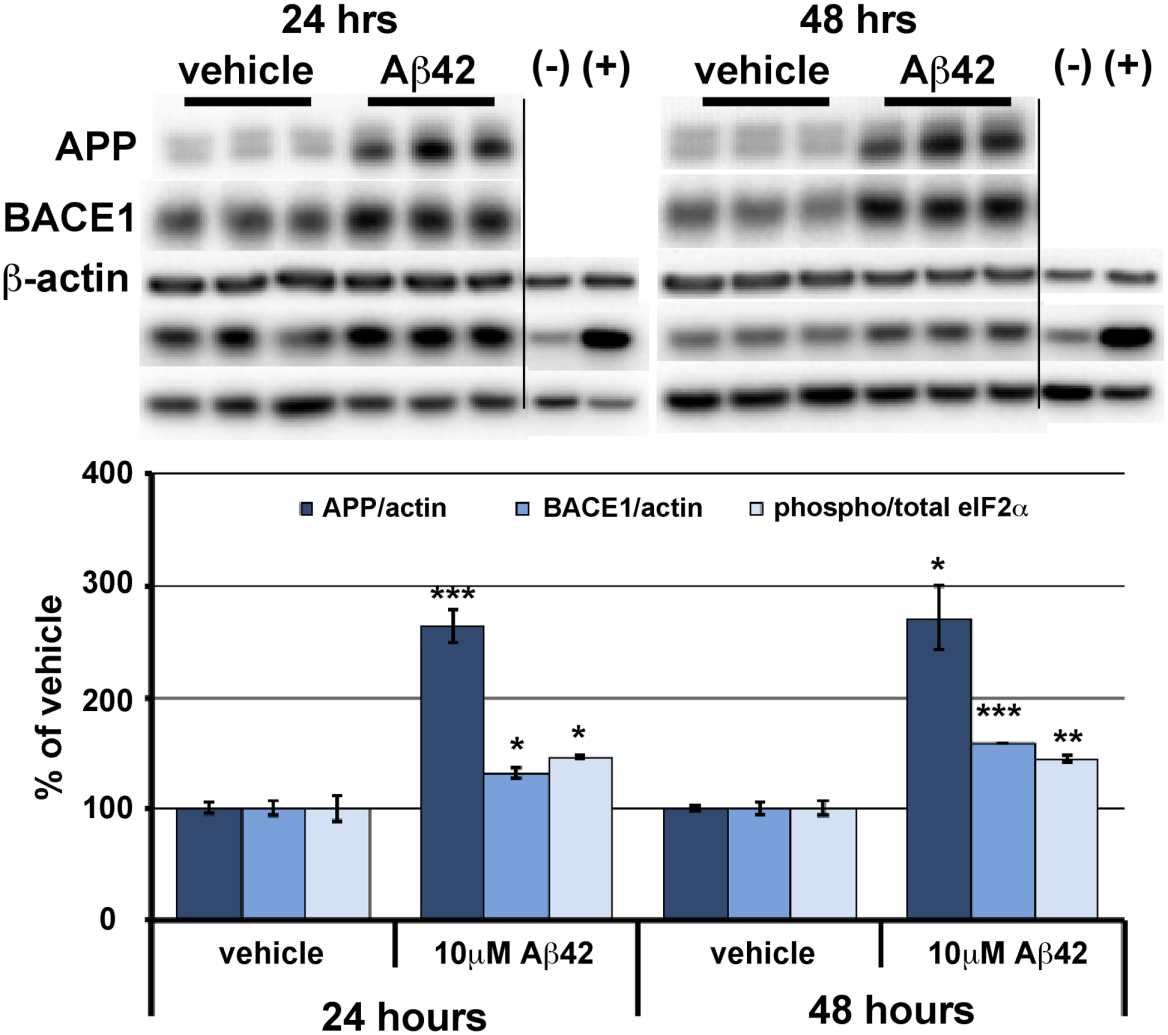
BACE1, APP, and phosphorylated eIF2α increase in response to A β42 oligomer treatment of primary neurons. Mixed cortical primary neurons were isolated from e15.5 mouse embryos, and after 7 days in culture, exposed to 10 µM oligomeric Aβ42 for 24 and 48 hrs. Cells were lysed in RIPA buffer and 10 µg/lane of protein were subjected to immunoblot analysis for APP, BACE1, phosphorylated (p)-eIF2α, total eIF2α, and β-actin as a loading control. (–) and (+) are negative and positive controls for eIF2α phosphorylation (control and UV treated HEK cell lysates, Cell Signaling). APP and BACE1 immunosignal intensities were normalized to those of β-actin. Phosphorylated and total eIF2α immunosignal intensities were measured and phosphorylated:total eIF2α ratio calculated. APP and BACE1 levels and phosphorylated:total eIF2α ratio (all measures displayed as percentage of vehicle control) are all significantly elevated by Aβ42 oligomer treatment at both time points. Bars represent SEM, n = 3 samples per condition, asterisks (*) indicate significant changes compared to respective vehicle, p<0.05*, p<0.01**, p<0.001***.

We previously reported that energy deprivation induces phosphorylation of the translation initiation factor eIF2α and that this in turn increases protein synthesis of the β-secretase enzyme, BACE1, in primary cultured neurons and in the brain [22]. Additionally, we found that levels of phosphorylated eIF2α (p-eIF2α) positively correlate with BACE1 levels and amyloid loads in human AD and APP transgenic mouse brains [22]. Furthermore, we observed that BACE1 levels increase in dystrophic presynaptic terminals that are in close proximity to amyloid plaques in AD and APP transgenic brains [24], [26] and that fibrillar and oligomeric Aβ42 cause BACE1 levels to increase ∼1.5–3-fold in primary neuron cultures [25]. Together, these results suggested the possibility that Aβ might induce eIF2α phosphorylation and cause an increase in BACE1 level via a translational mechanism similar to that of energy deprivation. To investigate this hypothesis, we established an in vitro culture system of Aβ42-induced BACE1 elevation in mouse primary cortical neurons (Fig. 1). Synthetic Aβ42 oligomers and fibrils [58] robustly elevate BACE1 levels in primary neuron cultures within 24 hrs, although Aβ42 oligomers appear more potent [25]. Therefore, we treated E15.5 mouse mixed cortical primary neuron cultures with 10 µM Aβ42 oligomers or vehicle for 24 and 48 hrs and then performed immunoblot analyses for levels of BACE1, p-eIF2α, and total eIF2α (Fig. 1). As expected, Aβ42 oligomers caused significant increases in BACE1 levels in both 24- and 48-hr treated primary neurons. Moreover, the p-eIF2α/total eIF2α ratio was also significantly elevated by Aβ42 oligomer compared to vehicle treatment. APP levels are also robustly increased by Aβ42, suggesting the intriguing possibility that Aβ42 induces elevation of both BACE1 and APP to initiate a pathogenic feed-forward cycle of accelerated Aβ42 generation. These results validated our primary neuron culture system for investigating the molecular mechanism of Aβ-induced BACE1 elevation and the potential involvement of eIF2α phosphorylation in this process.

To determine whether eIF2α phosphorylation mediates the Aβ-induced increase of BACE1 level in primary neurons in vitro, we undertook two genetic approaches to reduce p-eIF2α levels: Growth Arrest and DNA Damage protein 34 (GADD34)-mediated eIF2α de-phosphorylation [60] and the S51A targeted eIF2α mutant allele that prevents eIF2α phosphorylation [49]. In the first approach, we employed GADD34, a regulatory subunit of protein phosphatase 1c (PP1c) responsible for de-phosphorylating eIF2α at serine 51 following recovery from certain types of physiological stress [61]. The carboxy-terminal region of GADD34 activates PP1c, but normally this interaction is inhibited by the amino-terminal domain of GADD34. However, a GADD34 construct that lacks the inhibitory amino-terminal domain constitutively de-phosphorylates eIF2α [60]. Overexpression of this constitutively active GADD34 construct (GADD34 CA) greatly reduces levels of p-eIF2α, while a GADD34 lacking the carboxy-terminal activation domain (GADD34 control) has no effect on eIF2α phosphorylation and is frequently used as a negative control [60], [61].

**Figure 2:**
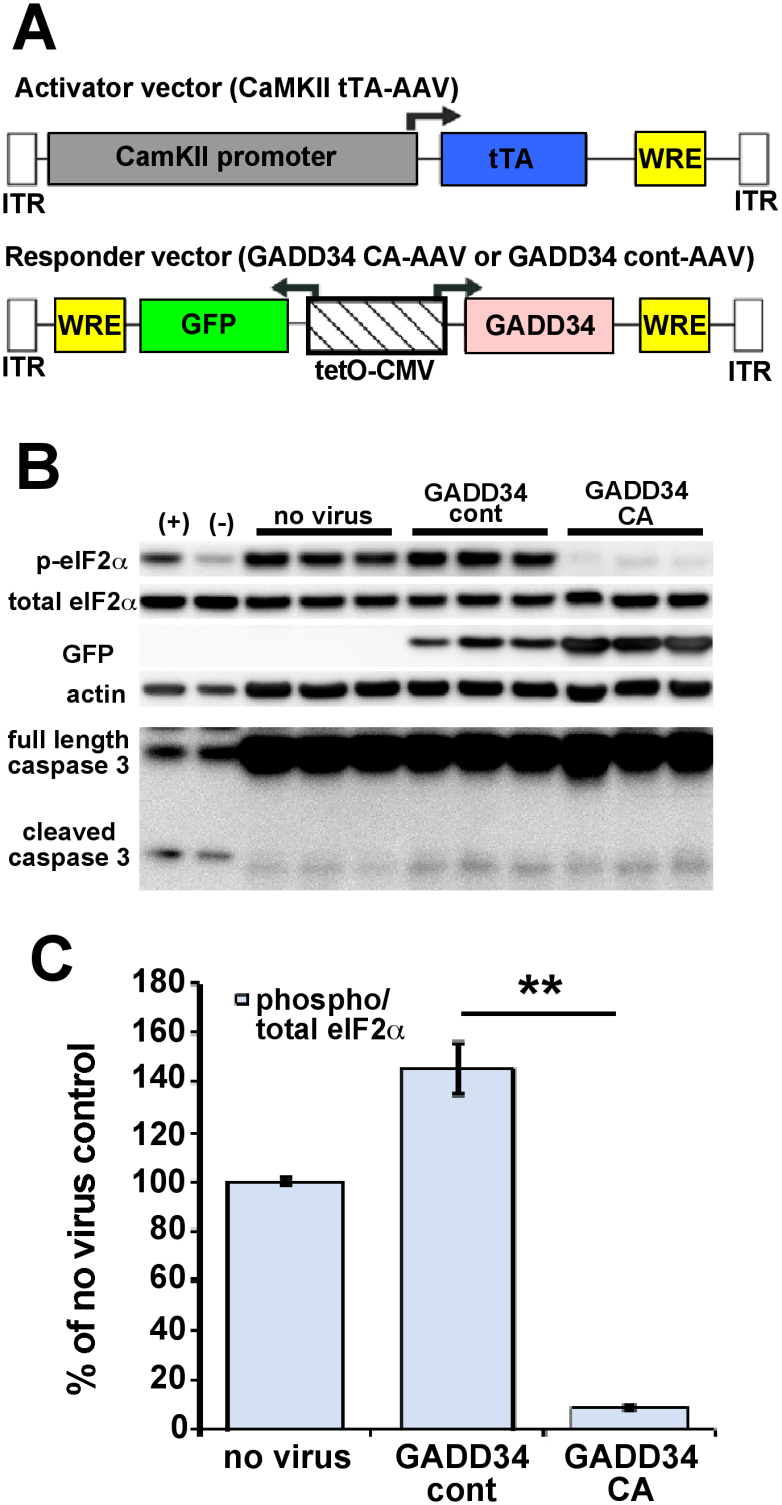
AAV vectors efficiently transduce primary neurons and block eIF2α phosphorylation with minimal toxicity. (A) A two-vector adeno-associated virus (AAV) system was used to decrease eIF2α phosphorylation specifically in excitatory forebrain neurons. The “activator” vector (CaMKII tTA-AAV) expresses the tetracycline transactivator (tTA) protein from a calmodulin kinase II (CaMKII) promoter, while the “responder” vector (GADD34 CA-AAV or GADD 34 cont–AAV) expresses GFP and either GADD34 constitutive active (CA) or GADD34 control (cont) from a bi-directional tetracycline operator-CMV promoter (tetO-CMV). All expressed transgenes contain a woodchuck post-trascriptional regulatory element (WRE) to enhance expression. ITR  =  inverted terminal repeat sequences. (B) Mixed cortical primary neurons were isolated from e15.5 mouse embryos and infected with 1×10^7^ viral genomes of CaMKII tTA-AAV and 1×10^7^ viral genomes of GADD34 CA-AAV or GADD 34 cont–AAV per well of a 12 well plate on the day of isolation. No virus was added to other wells as a negative control. Media was changed 48 hours later. After 8 days in culture, neurons were lysed for immunoblot analysis for total eIF2α, phosphorylated (p)-eIF2α, GFP, caspase 3 (full length and activated cleaved 17 kDa fragment), and β-actin as a loading control. (+) are HEK293 cells treated 30 minutes with 1 µM thapsigargin as a positive control for eIF2α phosphorylation, while (–) are untreated HEK293 cells as a negative control. Note that AAV transduction does not cause toxicity as indicated by minimal cleaved caspase 3 fragment. (C) Phosphorylated and total eIF2α immunosignal intensities were measured from the blot in (B) and phosphorylated:total eIF2α ratio (phospho/total eIF2α) was calculated and displayed as percentage of no virus control. Primary neurons transduced with GADD34 CA-AAV had dramatically less eIF2α phosphorylation than neurons transduced with GADD34 cont-AAV or no virus, as demonstrated by phospho/total eIF2α ratio. Note that GADD34 cont-AAV transduction caused a significant elevation of phospho/total eIF2α ratio compared to no virus control. Bars represent SEM, n = 3 samples per condition, p<0.01**.

We generated adeno-associated virus (AAV) “responder” vectors that co-express GADD34 CA or GADD34 control (GADD34 CA-AAV and GADD34 control-AAV, respectively) along with green fluorescent protein (GFP) under the control of a bi-directional tetO-CMV fusion promoter [50] (Fig. 2A). The transcription of GADD34 CA and GADD34 control is activated in the presence of a second “activator” AAV vector that expresses the tetracycline transactivator (tTA) under the control on the forebrain excitatory neuron-specific calmodulin kinase II promoter (CaMKII tTA-AAV). Addition of the tetracycline analog, doxycycline, inactivates GADD34 CA or GADD34 control transcription. However, because we did not use doxycycline, GADD34 CA or GADD34 control transcription was constitutive in these experiments. GADD34 CA-AAV or GADD34 control-AAV was added together with CaMKII tTA-AAV to E15.5 mouse mixed cortical primary neurons. The media was changed after 48 hours and the neurons were harvested at 7 days in vitro and subjected to immunoblot analysis (Fig. 2B). Notably, eIF2α phosphorylation was dramatically decreased in the primary neurons transduced with GADD34 CA AAV compared to GADD34 control-AAV, which caused a modest increase in p-eIF2α level (Fig. 2C). Transduction with either AAV vector resulted in only a small increase in pro-apoptotic cleaved caspase 3 (17 kDa) fragment. These results demonstrated that GADD34 CA AAV could effectively transduce primary neurons with minimal toxicity and cause a significant reduction in p-eIF2α level.

**Figure 3:**
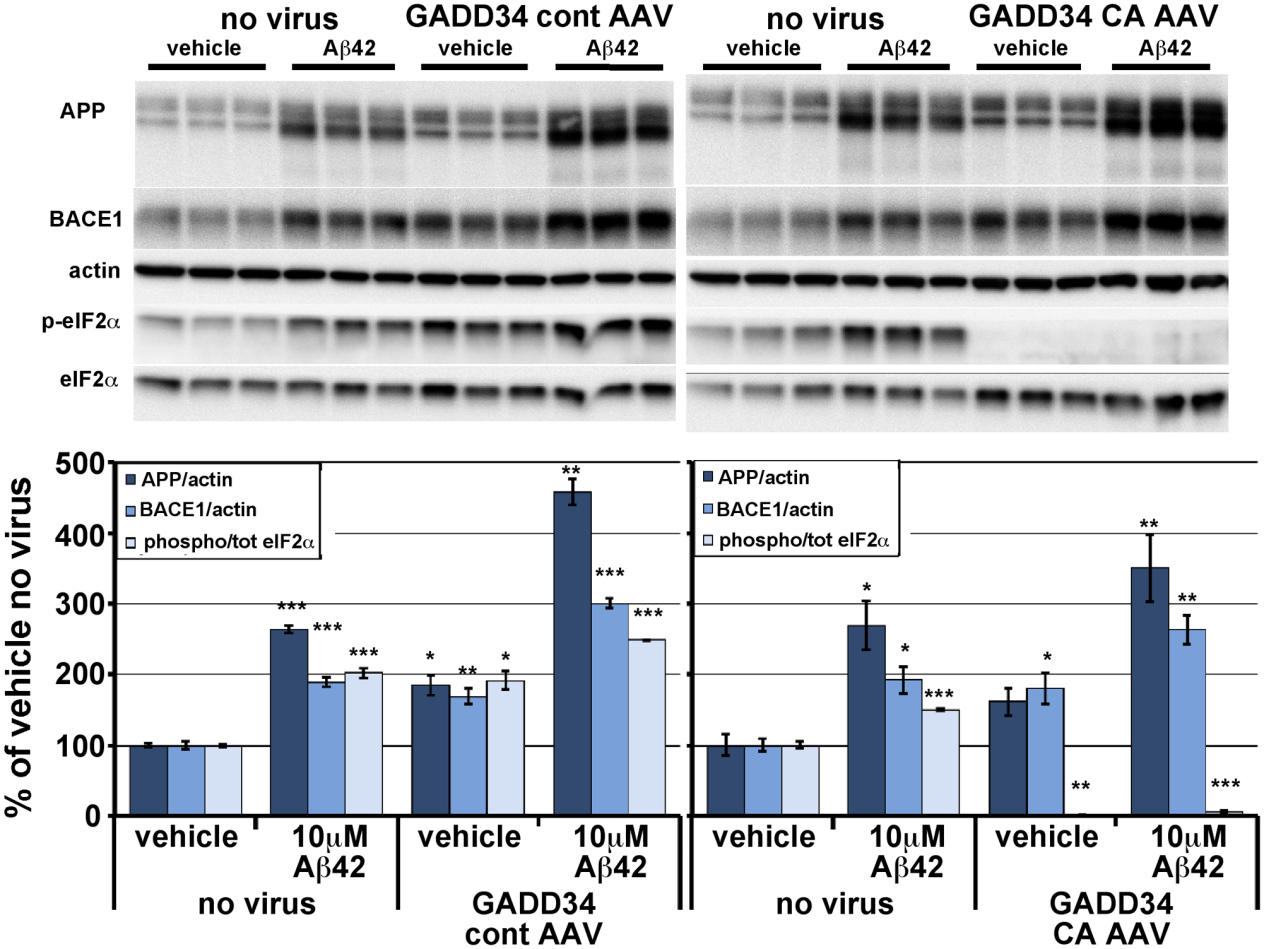
In primary neurons, GADD34 CA-AAV mediated reduction of eIF2α phosphorylation does not inhibit BACE1 and APP elevation in response to Aβ42 oligomer treatment. Mixed cortical primary neurons were isolated from e15.5 mouse embryos and infected with 1×10^7^ viral genomes CaMKII tTA-AAV and 1×10^7^ viral genomes of GADD34 CA-AAV or GADD 34 cont–AAV per well of a 12 well plate on the day of isolation. No virus was added to other wells as a negative control. Media was changed 48 hours later. After 7 days in culture, neurons were treated with vehicle or 10 µM Aβ42 oligomers generated as described [58], [59], lysed 30 hours later, and 10 µg/lane of protein were subjected to immunoblot analysis for APP, BACE1, total eIF2α, phosphorylated (p)-eIF2α, and β-actin as a loading control. APP and BACE1 immunosignal intensities were normalized to those of β-actin. Phosphorylated and total eIF2α immunosignal intensities were measured and phosphorylated:total eIF2α (phospho/total eIF2α) ratio calculated. All measures are displayed as percentage of no virus vehicle control. Note that levels of APP, BACE1 and p-eIF2α were significantly elevated by either Aβ42 oligomer treatment or GADD34 cont-AAV transduction alone, compared to no virus vehicle control. Similarly, GADD34 CA-AAV transduction alone also elevated APP and BACE1 levels but completely abrogated eIF2α phosphorylation. Interestingly, GADD34 cont-AAV transduction plus Aβ42 treatment increased APP and BACE1 levels and phospho/total eIF2α ratio to even greater extents than either treatment alone. GADD34 CA-AAV transduction plus Aβ42 treatment also elevated APP and BACE1 levels significantly, despite reducing the phospho/total eIF2α ratio to only ∼7% of no virus vehicle control. Bars represent SEM, n = 3 samples per condition, asterisks (*) indicate significant changes compared to “vehicle no virus”, p<0.05*, p<0.01**, p<0.001***.

Next, we sought to determine whether GADD34 CA AAV-mediated reduction of eIF2α phosphorylation could inhibit the Aβ42-induced BACE1 increase in primary neurons. Primary neuron cultures were transduced with GADD34 CA-AAV or GADD34 control-AAV for 48 hrs as described above, then 7 days later were treated with 10 µM Aβ42 oligomers for 30 hrs. Aβ42 oligomers were prepared from HFIP lyophilized recombinant Aβ42, as described previously [43], [51]. Following Aβ42 treatment, primary neurons were lysed in RIPA buffer and subjected to immunoblot analysis (Fig. 3). As expected, in the absence of either AAV vector, Aβ42 oligomer treatment caused a ∼2-fold increase of both BACE1 level and the ratio of phosphorylated to total (p:t) eIF2α in primary neurons. Interestingly, transduction with GADD34 control-AAV alone significantly elevated BACE1 level and p:t eIF2α ratio. Likewise, GADD34 CA-AAV alone also increased BACE1 level but completely abolished eIF2α phosphorylation, indicating an eIF2α phosphorylation independent mechanism of Aβ42-induced BACE1 elevation. Combined Aβ42 treatment plus GADD34 control-AAV transduction caused ∼3-fold and ∼2.5-fold increases in BACE1 level and p:t eIF2α ratio, respectively, even higher than Aβ42 oligomers alone, suggesting an additive Aβ42-AAV effect. Combined Aβ42 treatment plus GADD34 CA-AAV transduction of primary neurons resulted in a ∼2.5 fold increase in BACE1 level but nearly completely abrogated eIF2α phosphorylation. Interestingly, APP levels were robustly increased by Aβ42 and both AAV vectors, either in single treatment or in combination with Aβ42. It is notable that the combination of Aβ42 plus either AAV vector most potently elevated BACE1 and APP levels. Taken together, these results suggest that Aβ42 is capable of elevating BACE1 and APP levels via a mechanism that does not depend upon phosphorylation of eIF2α, at least in primary neurons.

### The eIF2α S51A knockin mutation blocks eIF2α phosphorylation but does not prevent Aβ42-induced BACE1 elevation in cultured mouse cortical primary neurons

**Figure 4:**
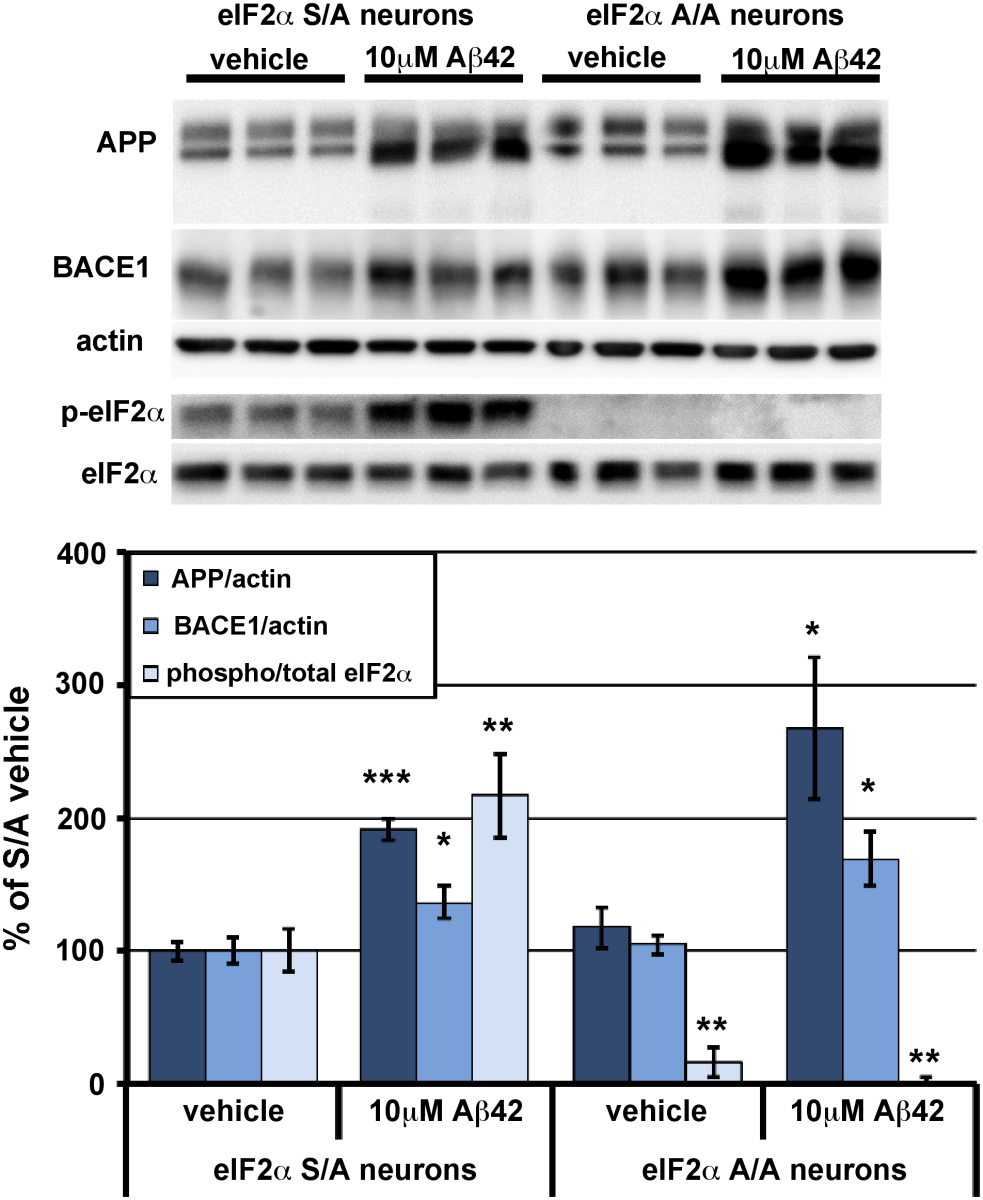
In primary neurons, genetic reduction of eIF2α phosphorylation via eIF2α S51A knockin mutation does not inhibit BACE1 and APP elevation in response to Aβ42 oligomer treatment. Mixed cortical primary neurons were isolated from e15.5 mouse embryos that were either homozygous for the eIF2α S51A targeted replacement mutation (A/A) or heterozygous (S/A). After 7 days in culture, neurons were treated with vehicle or 10 µM Aβ42 oligomers, lysed 30 hours later, and 10 µg/lane of protein were subjected to immunoblot analysis for APP, BACE1, total eIF2α, phosphorylated (p)-eIF2α, and β-actin as a loading control. APP and BACE1 immunosignal intensities were normalized to those of β-actin. Phosphorylated and total eIF2α immunosignal intensities were measured and phosphorylated:total eIF2α (phospho/total eIF2α) ratio calculated. All measures are displayed as percentage of S/A vehicle control. As expected, levels of APP, BACE1, and p-eIF2α were significantly elevated by Aβ42 oligomer treatment in S/A neurons, compared to vehicle. Importantly, Aβ42 oligomers caused robust increases of BACE1 and APP levels in A/A neurons, despite complete abrogation of eIF2α phosphorylation. There was no significant difference in BACE1 or APP levels in A/A compared to S/A neurons, eliminating the problem that AAV treatment of primary neurons increases levels of BACE1, APP, and p-eIF2α. The small phospho/total eIF2α ratio in vehicle treated A/A neurons is due to immunoblot background. Bars represent SEM, n = 6 samples per condition, asterisks (*) indicate significant changes compared to S/A vehicle, p<0.05*, p<0.01**.

To confirm our results that reducing the phosphorylation of eIF2α using GADD34 CA-AAV does not prevent Aβ42-induced BACE1 elevation in primary neurons, we turned to a different genetic model to block eIF2α phosphorylation: eIF2α S51A knockin mouse primary neurons. S51A targeted replacement mice have an alanine in place of the serine 51 that becomes phosphorylated under certain conditions of physiological stress; consequently, eIF2α cannot be phosphorylated in these animals [49]. Homozygous (A/A) eIF2α S51A knockin mice die soon after birth as a result of hypoglycemia, but heterozyotes (S/A) are viable and fertile [49]. Thus, we were able to obtain primary mixed cortical neuron cultures from E15.5 embryos of S/A×S/A mouse crosses. Primary neurons were harvested from the cortex of each embryo individually to obtain pure A/A and S/A cultures. After 7DIV, primary neurons were treated with 10 µM oligomeric Aβ42 for 30 hrs, then were harvested and subjected to immunoblot analysis (Fig. 4). As with wild-type primary neurons, Aβ42 oligomer treatment caused BACE1 level and p:t eIF2α ratio to increase significantly in S/A neuron cultures, compared to vehicle treated S/A cultures. Importantly, A/A primary neurons showed a similar Aβ42-induced increase in BACE1 level as S/A neurons even though eIF2α phosphorylation was completely abrogated in Aβ42 treated A/A neurons. Once again, APP levels were robustly increased by Aβ42 treatment in both S/A and A/A neuron cultures. Therefore, as with GADD34 CA-AAV transduced primary neurons treated with Aβ42, our results with A/A neurons indicate that a mechanism other than eIF2α phosphorylation is responsible for Aβ42-induced BACE1 and APP elevation in primary neurons.

### Overexpression of constitutively active GADD34 inhibits eIF2α phosphorylation but does not reduce BACE1 elevation in 5XFAD transgenic mouse brain

**Figure 5:**
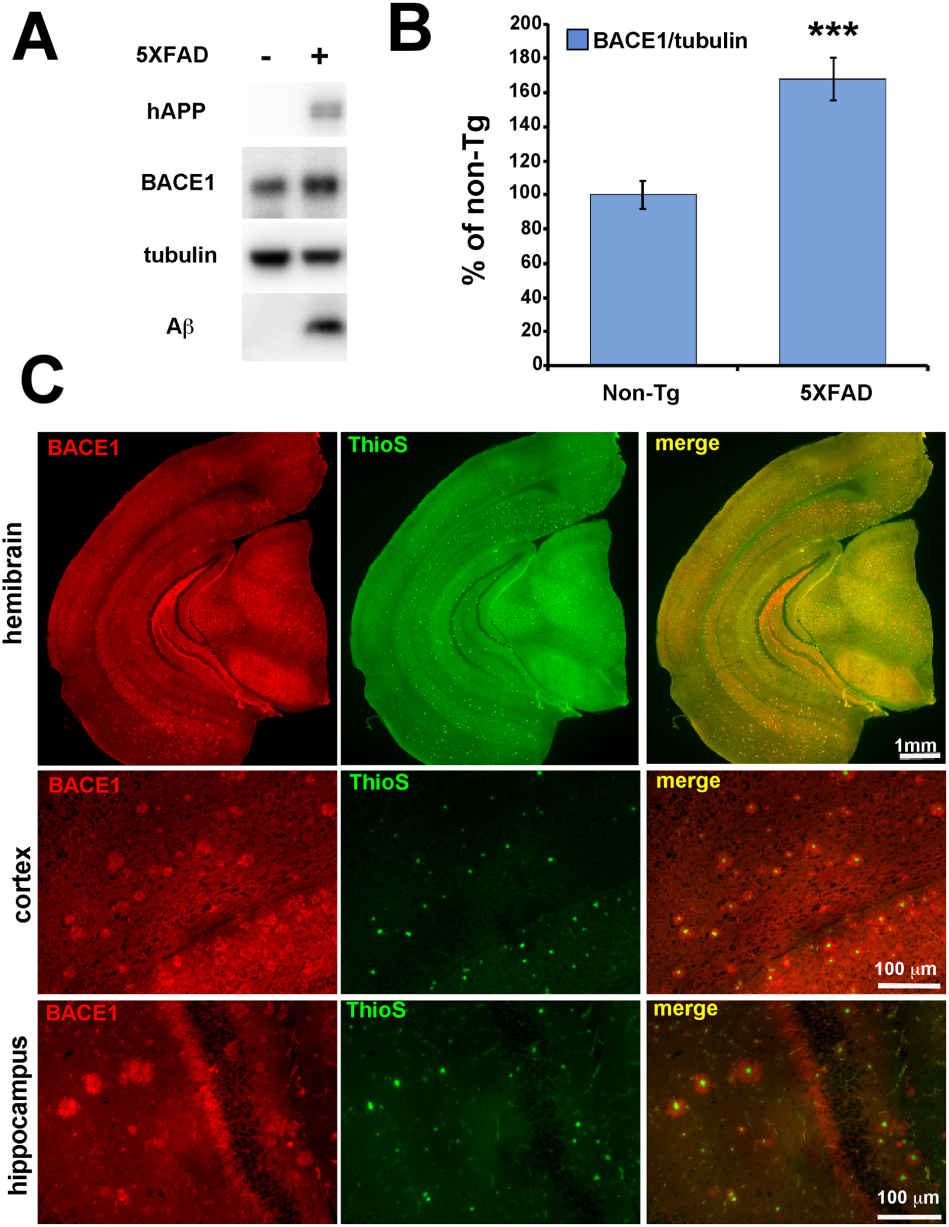
BACE1 is elevated in 5XFAD transgenic mouse brain, with highest concentrations surrounding amyloid plaques. (A) Hemibrains from 6 month old 5XFAD (+) mice (n = 17), and non-transgenic (Tg) (–) age-matched controls (n = 13) were homogenized and 20 µg/lane of protein were subjected to immunoblot analysis for transgenic human (h) APP, BACE1, Aβ, and βIII-tubulin as a loading control. (B) hAPP and BACE1 immunosignal intensities were normalized to those of βIII-tubulin and displayed as percentage of non-Tg control. Note that 5XFAD mice have significantly elevated levels of BACE1 and Aβ compared to non-Tg controls, as detected by BACE1 antibody clone 3D5 and human APP/Aβ antibody clone 6E10, respectively. Bars represent SEM, asterisks (*) indicate significant changes compared to non-Tg control, p<0.001***, (C) Coronal brain sections from 5XFAD mice were co-stained with anti-BACE1 antibody (red) and Thioflavin S (green) for fibrillar amyloid and imaged by fluorescence microscopy. At low magnification, high levels of BACE1 (red) are readily observed in mossy fibers of the hippocampus, which is the normal localization pattern of BACE1 in the brain (BACE1, first row). At high magnification, BACE1 (red) is shown to concentrate abnormally in an annulus that immediately surrounds the fibrillar amyloid plaque core (green; cortex, second row; hippocampus, third row). Scale bars = 1 mm, first row; 100 µm, second and third rows.

Although our in vitro experiments definitively showed that eIF2α phosphorylation is not required for Aβ-induced BACE1 elevation, mature neurons in the brain are clearly different than cultured primary neurons in many respects and therefore might need phosphorylated eIF2α to increase BACE1 levels in response to amyloid plaques. To test this possibility, we used the two genetic approaches we employed before, GADD34 CA-AAV transduction and eIF2α S51A knockin mice, to reduce p-eIF2α levels in cerebral neurons of the 5XFAD transgenic (Tg) mouse model of amyloidosis [51]. This mouse line expresses two linked transgenes, both driven by the Thy1 promoter: 1) the 695 amino acid isoform of human APP with three famililal Alzheimer’s disease (FAD) linked mutations called Swedish (K670N, M671L) [62], Florida (I716V) [63] and London (V717I) [64]; 2) human PS1 with the two FAD mutations M146L and L286V [65]. These five mutations result in massive overproduction of Aβ42, intraneuronal Aβ42 accumulation, plaque deposition at 2 months, memory impairment at 4–5 months, and neuron loss starting at 6 months [51], [66]. 5XFAD mice rapidly develop florid amyloid pathology. Additionally, BACE1 levels are globally increased ∼1.5-fold in 5XFAD brain, with the vast majority of this BACE1 elevation being concentrated in dystrophic neurites that are in close proximity to amyloid plaques (Fig. 5; [24], [26]). Moreover, previous work had indicated that both phosphorylated and total eIF2α levels were elevated in 5XFAD brain at 6 months, leading to a small but significant increase in the p:t eIF2α ratio [22]. Thus, we reasoned that the 5XFAD mouse is an appropriate model for investigating the potential role of eIF2α phosphorylation in BACE1 elevation near amyloid plaques.

First, we transduced GADD34 CA-AAV into the brains of 5XFAD mice using the somatic brain transgenesis technique, which results in expression of the AAV vector transgene throughout the brain for the life of the mouse [53], [67], [68]. High-titer AAVs were bilaterally injected into the lateral ventricles of post-natal day 0 (P0) pups within hours of birth. In a pilot experiment, immunoblot analysis of homogenates from control AAV-injected hemibrains harvested at 1 and 3 months of age showed that AAV transduction did not elevate BACE1 or p-eIF2α levels in the brain (data not shown), in contrast to cultured primary neurons, indicating that AAV on its own does not appear to perturb the expression, regulation, or metabolism of these proteins in vivo.

**Figure 6:**
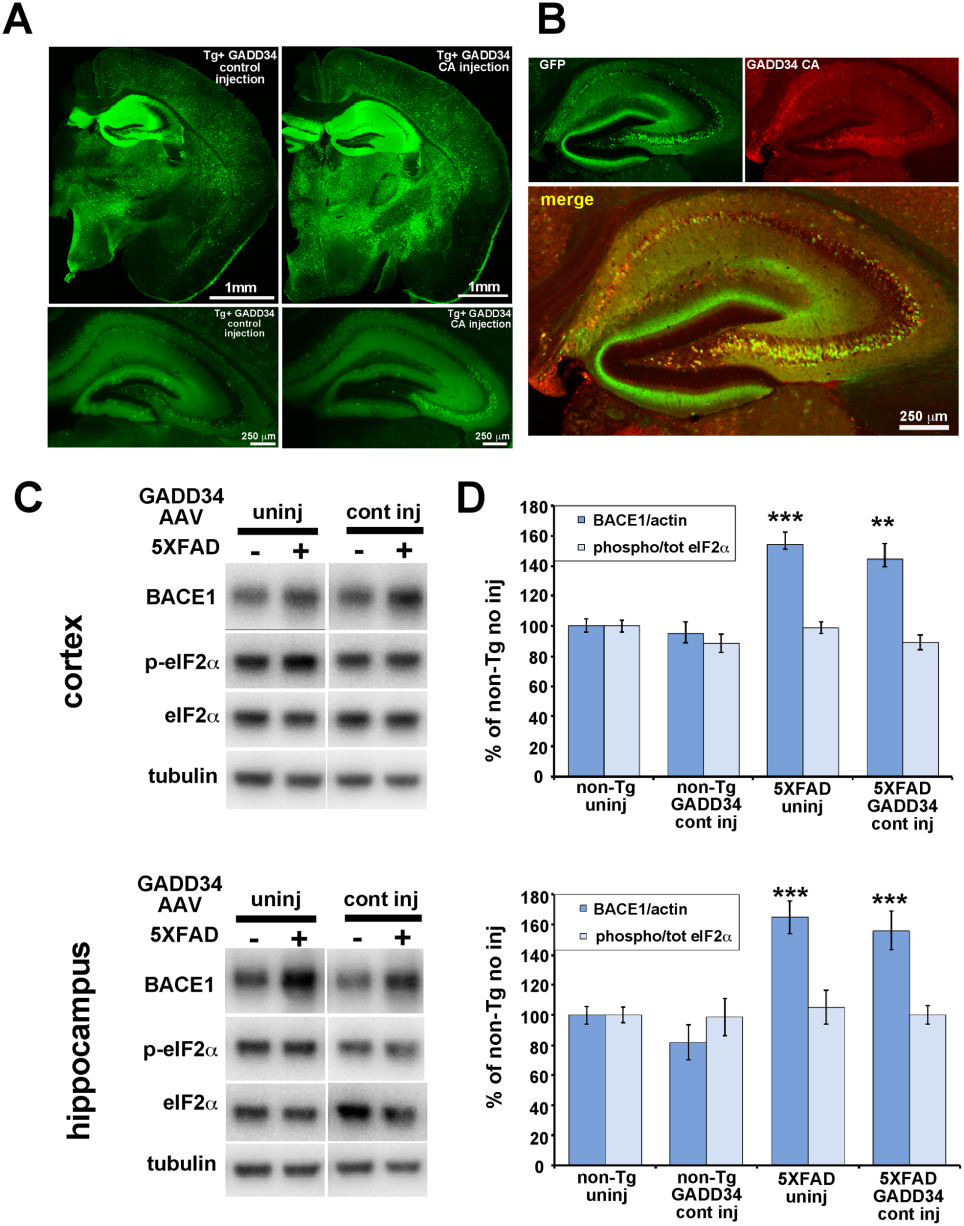
Two-vector AAV system effectively transduces mouse brain, and GADD34 control AAV transduction does not elevate levels of BACE1 or phosphorylated eIF2α in the brain. 5XFAD or non-Tg pups were injected on postnatal day 0 into lateral ventricles with 2 µl per hemisphere containing 6.6×10^10^ viral genomes of GADD34 CA-AAV or GADD 34 cont–AAV plus 6.9×10^10^ viral genomes of CaMKII tTA-AAV. Mice were aged to 6 months and brains harvested for immunoblot, and immunofluorescence microscopy analysis. (A) GFP fluorescence in coronal brain sections of 6 month-old 5XFAD mice injected with GADD 34 cont–AAV (left column) or GADD34 CA-AAV (right column) shows comparable expression levels of GFP from both transduced AAV vectors. Upper row: entire hemibrain sections showing wide-spread GFP expression, especially in the hippocampus. Lower row: lower exposure of hippocampus showing cellular GFP expression. The AAV serotype 1 with the CaMKII promoter effectively drives expression in excitatory forebrain neurons, with particularly high expression in the hippocampus. Scale bar  =  1 mm (top row), 250 µm (bottom row). (B) Low exposure high magnification image of a section of the hippocampus from a mouse transduced with GADD34 CA-AAV stained with an antibody against GADD34 (red) shows high co-localization of GADD34 CA and GFP in neurons of the CA regions, indicating that GFP fluorescence is an effective proxy marker for GADD34 expression Scale bar = 250 µm. (C) 20 µg/lane of cortex or hippocampus homogenate from 6 month-old 5XFAD (+) and non-Tg (–) mice either uninjected (uninj) or GADD 34 cont–AAV injected (cont inj) were subjected to immunoblot analysis for BACE1, total eIF2α, phosphorylated (p)-eIF2α, and βIII-tubulin as a loading control. All samples were transferred onto a single piece of PVDF membrane, as described in Methods, and representative blots are shown here. (D) BACE1 immunosignal intensities were normalized to those of βIII-tubulin. Phosphorylated and total eIF2α immunosignal intensities were measured and phosphorylated:total eIF2α (phospho/total eIF2α) ratio calculated. All measures are displayed as percentage of uninjected non-Tg control. Comparison of GADD34 cont–AAV injected mice with genotype-matched uninjected mice shows that there is no effect on BACE1 or p-eIF2α levels from AAV brain injection itself. n = 9–15 mice per group. Bars represent SEM. Asterisks (*) indicate significant difference from non-Tg uninj p<0.01**, p<0.001***.

Next, we injected GADD34 CA-AAV or GADD34 control-AAV, together with the activator CamKII-tTA AAV, into the ventricles of P0 5XFAD and non-Tg pups, allowed mice to age to 6 months, and then harvested cortices and hippocampi for biochemical and histological analyses of BACE1, p-eIF2α, and Aβ42. Importantly, GADD34 CA-AAV and GADD34 control-AAV vectors were able to transduce and express at similar levels in the brain as indicated by GFP fluorescence in brain sections (Fig. 6A) and GFP immunoblot analysis of brain homogenates (data not shown). Brain sections immunostained with an antibody against GADD34 showed that GADD34 CA co-localized well with GFP (Fig. 6B), suggesting that GFP fluorescence is a good proxy for GADD34 transgene expression. Both AAVs displayed a neuronal expression pattern, as expected for the CaMKII promoter, with highest levels in the hippocampus followed by the cortex. Immunoblot analysis of homogenates from 6 month old hippocampi and cortices showed no difference in BACE1 or p-eIF2α level in hippocampus or cortex between uninjected and GADD34 control-AAV injected mice of either non-Tg or 5XFAD genotypes (Fig. 6C, D). This results confirms our previous pilot experiment showing that AAV on its own does not affect BACE1 or p-eIF2α levels in the brain, in contrast to AAV transduction of primary neurons in culture (Figs. 2, 3). As expected, BACE1 levels were significantly increased in 5XFAD compared to non-Tg brains, irrespective of AAV injection (Fig. 6C, D). However, p:t eIF2α ratio was not elevated by the presence of the 5XFAD transgene, contrary to our previous observation [22]. The reason for the absence of an increased p:t eIF2α ratio in 5XFAD brain is unclear and is currently under investigation.

**Figure 7:**
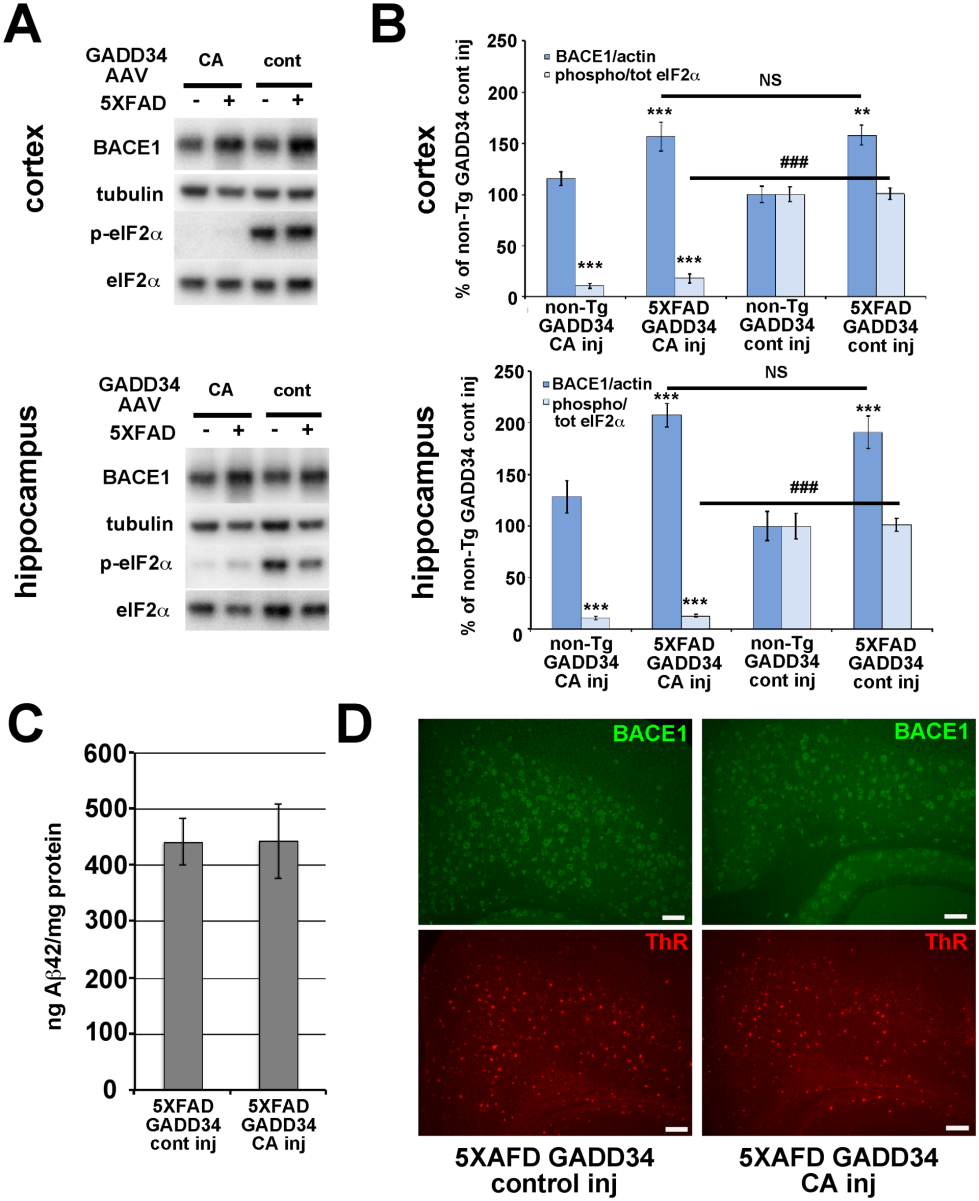
GADD34 CA-AAV effectively inhibits eIF2α phosphorylation in 5XFAD brain but does not block amyloid-associated BACE1 elevation. 5XFAD or non-Tg pups were injected on postnatal day 0 into lateral ventricles with 2 µl per hemisphere of 6.6×10^10^ viral genomes of GADD34 CA-AAV or GADD 34 cont–AAV plus 6.9×10^10^ viral genomes of CaMKII tTA-AAV. Mice were aged to 6 months and brains harvested for immunoblot and immunofluorescence microscopy analysis. (A) 20 µg/lane of cortex or hippocampus homogenate from 6 month-old 5XFAD (+) and non-Tg (–) mice either GADD34 CA-AAV injected (CA) or GADD 34 cont–AAV injected (cont) were subjected to immunoblot analysis for BACE1, total eIF2α, phosphorylated (p)-eIF2α, and βIII-tubulin as a loading control. All samples were transferred onto a single piece of PVDF membrane, as described in Methods, and representative blots are shown here. (B) BACE1 immunosignal intensities were normalized to those of βIII-tubulin. Phosphorylated and total eIF2α immunosignal intensities were measured and phosphorylated:total eIF2α (phospho/total eIF2α) ratio calculated. All measures are displayed as percentage of GADD 34 cont–AAV injected non-Tg control. BACE1 levels were elevated in GADD 34 cont–AAV transduced 5XFAD cortex and hippocampus compared to non-Tg, as expected. Importantly, GADD34 CA-AAV transduction reduced p-eIF2α levels by ∼85–90% compared to GADD34 cont-AAV transduction in both 5XFAD and non-Tg cortex and hippocampus. Despite this dramatic inhibition of eIF2α phosphorylation, BACE1 levels were elevated in GADD34 CA-AAV transduced 5XFAD cortex and hippocampus to the same extent as in GADD34 cont-AAV transduced 5XFAD cortex and hippocampus. n = 6–15 mice per group, bars represent SEM, asterisks (*) indicate significant changes compared to non-Tg GADD34 cont-AAV control, NS  =  not significant, p<0.05*, p<0.01**, p<0.001***, (#) represents significant difference between 5XFAD GADD34 cont-AAV and 5XFAD GADD34 CA-AAV p<0.001 ###. (C) Cortical homogenates from 5XFAD mice injected with either GADD 34 cont–AAV or GADD34 CA-AAV were prepared for measurement of total (soluble plus insoluble) Aβ42 levels (ng/mg total protein) by ELISA (Methods). No significant difference in total Aβ42 level between GADD34 CA-AAV and GADD34 cont-AAV brain transduction was observed. Bars represent SEM (D) Coronal brain sections of representative GADD34 CA-AAV or GADD34 cont-AAV transduced 5XFAD mice co-stained with anti-BACE1 antibody (green) and thiazine red (ThR) for fibrillar amyloid, then imaged by fluorescence microscopy. Both the intensities of BACE1 immunostaining and fibrillar plaque load signal appear unaffected by reduction of eIF2α phosphorylation via GADD34 CA-AAV transduction, thus corroborating our immunoblot analysis that phosphorylated eIF2α does not mediate amyloid-associated BACE1 elevation. Each image is taken at 10x objective magnification, at the same exposure, from the cortex just above hippocampal region CA3. Scale bar = 100 µm.

We next sought to determine the effects of GADD34 CA-AAV on BACE1 and p-eIF2α levels in brains of 6 month old 5XFAD mice (Fig. 7). Immunoblot analysis for p-eIF2α revealed that GADD34 CA-AAV injection produced a very effective reduction (∼85–90%) of the p:t eIF2α ratio in the hippocampus and cortex of both non-Tg and 5XFAD mice, compared to GADD34 control-AAV injection (Fig. 7A, B). Importantly, immunoblots showed that no significant difference in BACE1 level was observed between 5XFAD brains injected with GADD34 CA-AAV compared to GADD34 control-AAV, despite robust reduction of eIF2α phosphorylation for the former. Additionally, immunolabeling of 5XFAD brain sections with an anti-BACE1 antibody revealed no difference in the level of BACE1 immunostaining between GADD34 CA-AAV and GADD34 control-AAV injection (Fig. 7D). Moreover, Aβ42 level by ELISA (Fig. 7C) and amyloid plaque signal by thiazine red staining (Fig. 7D) of 5XFAD brain homogenates and sections, respectively, also showed no significant changes in response to GADD34 CA-AAV compared to GADD34 control-AAV injection. Taken together, these results demonstrate that amyloid-associated BACE1 elevation in the brain is not dependent upon the phosphorylation of eIF2α. Furthermore, reduction of eIF2α phosphorylation decreases neither Aβ42 level nor amyloid plaque pathology in vivo.

### eIF2α S51A knockin mutation inhibits eIF2α phosphorylation but does not reduce BACE1 elevation in 5XFAD transgenic mouse brain

**Figure 8:**
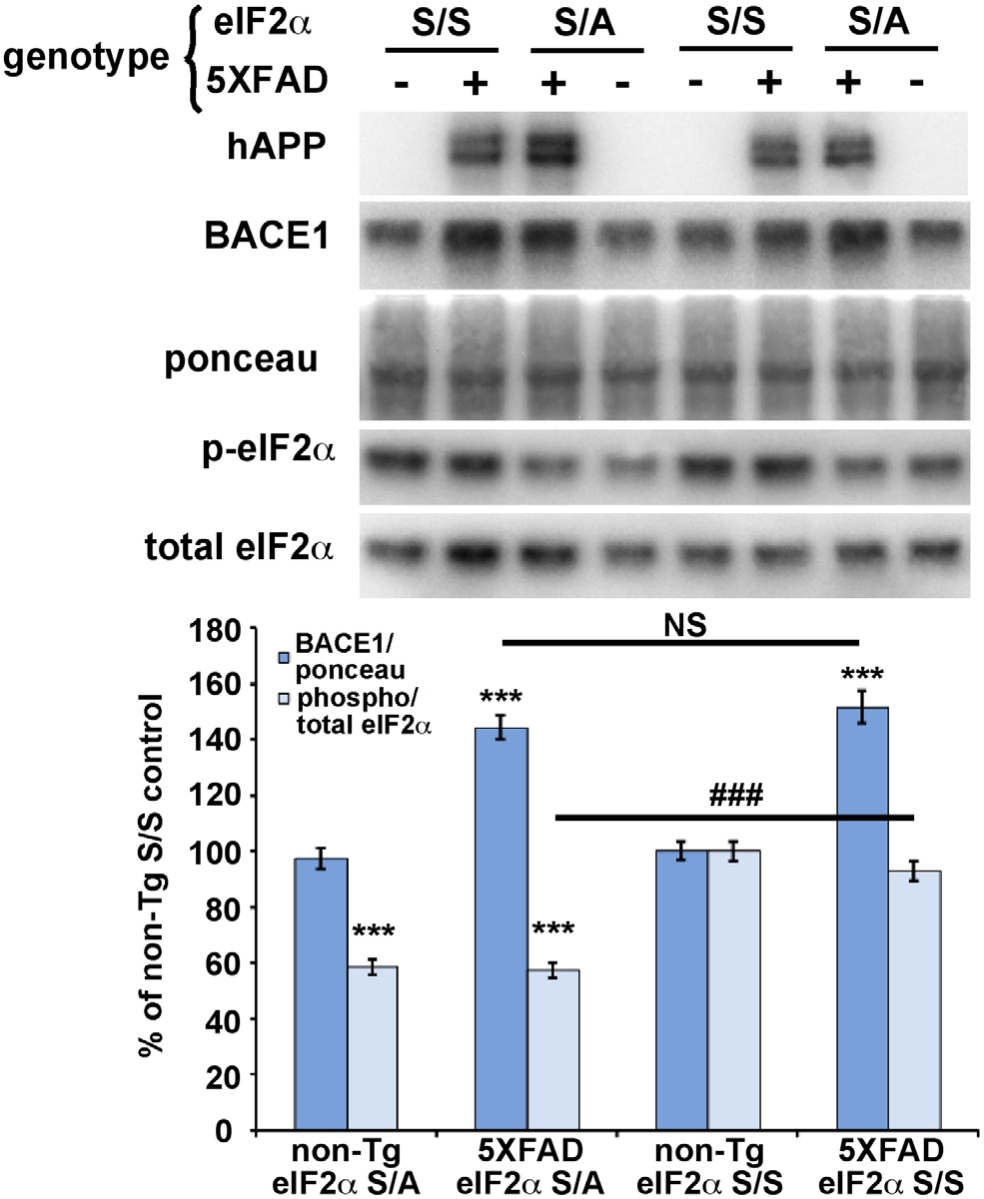
Genetic reduction of eIF2α phosphorylation via eIF2α S51A knockin mutation does not block amyloid-associated BACE1 elevation in 5XFAD brain. 5XFAD mice were crossed with mice harboring the eIF2α S51A targeted replacement mutation to generate 5XFAD (+) or non-Tg (–) offspring that were either heterozygous for the eIF2α S51A knockin mutation (S/A) or wild-type (S/S). Mice were aged to 12 months, brains harvested, and homogenates prepared. 20 µg/lane of brain homogenate were subjected to immunoblot analysis for transgenic human (h) APP, BACE1, total eIF2α, and phosphorylated (p)-eIF2α. All samples were transferred onto a single piece of PVDF membrane and stained with ponceau S as a protein loading control, as described in Methods. For quantification, BACE1 immunosignal intensity was normalized to ponceau S staining intensity for a given lane. Phosphorylated and total eIF2α immunosignal intensities were measured and phosphorylated:total eIF2α (phospho/total eIF2α) ratio calculated for a given lane. The means of each group were calculated and means displayed as percentage of the mean non-Tg S/S control. Both non-Tg and 5XFAD mice that were also heterozygous for the eIF2α S51A knockin mutation had a ∼40% reduction in phospho/total eIF2α ratio compared to their S/S counterparts; presumably, the phospho/total eIF2α ratios did not reach the expected 50% reduction because of a high non-specific background on the p-eIF2α immunoblot or partial compensatory increased phosphorylation of the wild type allele. Importantly, BACE1 level in 5XFAD; S/A brain showed an amyloid-associated elevation that was equivalent to that of 5XFAD; S/S brain, despite the 40% reduction in phospho/total eIF2α ratio. n = 19–30 mice per group. Bars represent SEM, asterisks (*) indicate significant changes compared to non-Tg S/S control, p<0.05*, p<0.01**, p<0.001***, NS  =  not significant, (#) indicates significant difference between 5XFAD S/S and 5XFAD S/A p<0.001 ###.

Our AAV results suggested that brain injection of viral vectors in general did not appear to affect the outcome of our experiments. However, to provide further confidence and additional support for our conclusions, we sought to reduce eIF2α phosphorylation by a different genetic method that did not involve virus injection. To do so, we crossed 5XFAD mice with heterozygous eIF2α S51A knockin mice to generate 5XFAD; eIF2α S/A bigenic offspring that have ∼50% reduction of eIF2α phosphorylation (5XFAD; S/A mice). Only S/A heterozygotes could be analyzed, since A/A homozygotes die shortly after birth, as reported previously [49]. Immunoblot analysis showed the expected increase in BACE1 level for 12 month old 5XFAD; S/S (wild-type) compared to non-Tg; S/S mice (Fig. 8). Importantly, we oberved that BACE1 level in 5XFAD; S/A brain was the same as that in 5XFAD; S/S brain, despite the fact that p:t eIF2α ratio was reduced by over 40%. Additionally, Aβ42 levels by ELISA were unchanged between 5XFAD; S/A and 5XFAD; S/S genotypes (data not shown). Once again, we could discern no significant effect of the 5XFAD transgene on the p:t p-eIF2α ratio compared to non-Tg mice, contrary to our previous report [22]. These results are consistent with those of our AAV experiments, and provide further support for the conclusion that eIF2α phosphorylation does not mediate amyloid-associated BACE1 elevation in the brain.

### BACE1-YFP expressed from a transgene lacking the BACE1 mRNA 5′ UTR is also elevated and accumulates around amyloid plaques in 5XFAD brain

**Figure 9:**
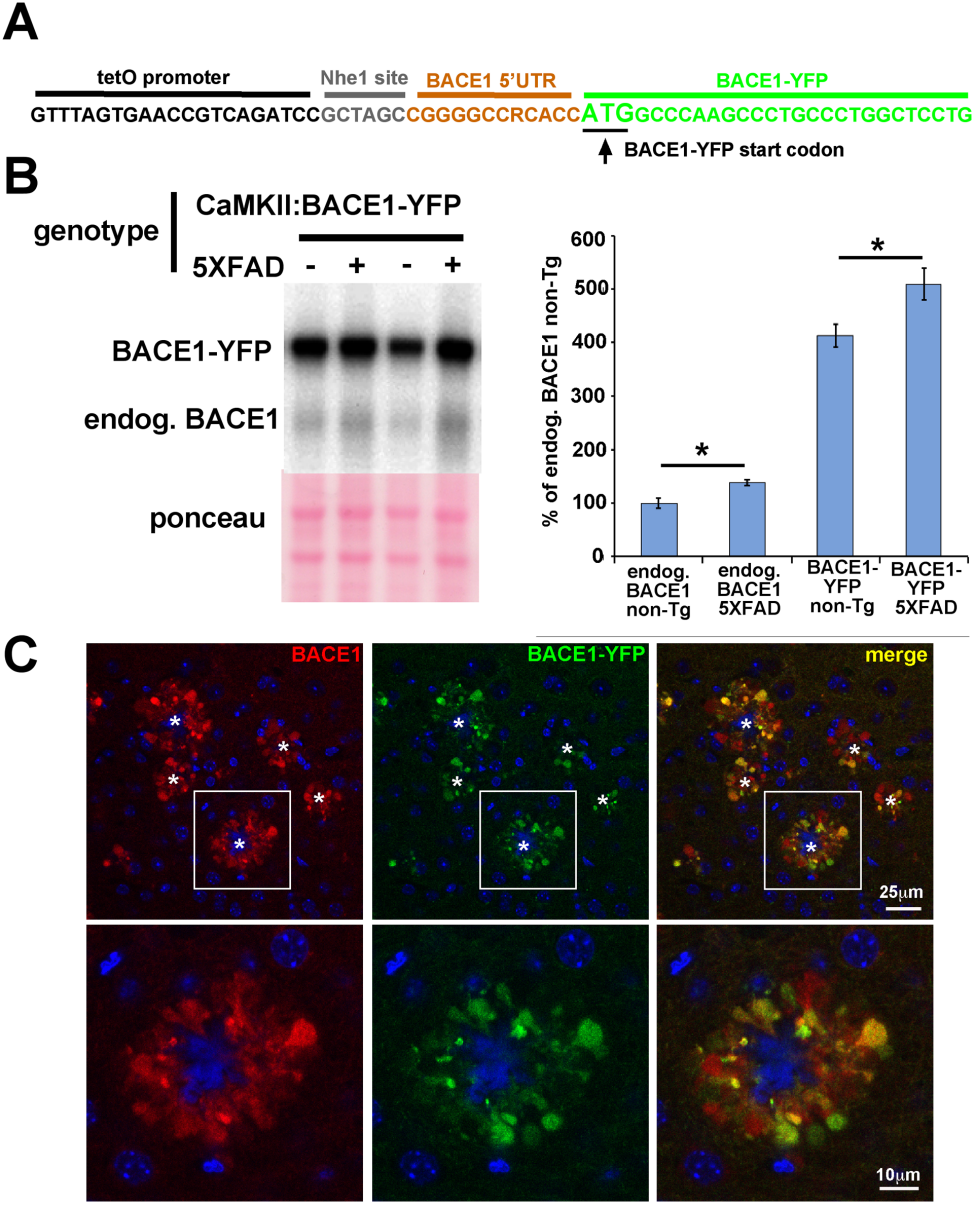
BACE1-YFP expressed from a transgene with a truncated BACE1 mRNA 5′ UTR is also elevated and accumulates around amyloid plaques in 5XFAD brain. (A) 5′ UTR of BACE1-YFP transgene. The BACE1-YFP coding region (green) was subcloned into the tetO promoter vector PMM400 (black) via an NheI site (gray) [52], leaving a severely truncated BACE1 mRNA 5′ UTR (orange) consisting of only eleven nucleotides that lack the uORFs required for de-repression of translation by phosphorylated eIF2α. (B) 5XFAD mice were crossed with BACE1-YFP transgenic mice to generate 5XFAD (+) and non-Tg (–) offspring that also expressed the BACE1-YFP transgene. 5XFAD and non-Tg offspring that lacked the BACE1-YFP transgene were also generated. At 6–8 months of age, cortices of 5XFAD; BACE1-YFP, non-Tg; BACE1-YFP, 5XFAD, and non-Tg littermates (n = 5 for each group) were harvested, homogenized, and 20 µg/lane of homogenates were subjected to immunoblot analysis for BACE1 using the 3D5 anti-BACE1 antibody. The immunoblot was stained with ponceau S as a protein loading control. Representative BACE1-YFP immunoblot signals are shown. BACE1-YFP runs at ∼90 kDa on SDS-PAGE, compared to ∼65 kDa for endogenous (endog.) BACE1. For quantification, BACE1 and BACE1-YFP immunosignal intensities were normalized to ponceau S staining intensity for a given lane. The means of each group were calculated and means displayed as percentage of the mean BACE1 level in non-Tg control. The BACE1-YFP transgene is expressed at levels that are ∼4-fold higher than that of endogenous BACE1. As expected, endogenous BACE1 level is significantly elevated in 5XFAD brain compared to non-Tg brain. Most importantly, BACE1-YFP levels also exhibit a significant amyloid-associated elevation with the 5XFAD genotype compared to the non-Tg genotype, despite the complete absence of uORFs necessary for regulation by eIF2α phosphorylation. Bars represent SEM, asterisks (*) indicate significant changes compared to respective non-Tg control, p<0.05*. (C) Sagittal section of representative 5XFAD; BACE1-YFP cortex stained with anti-BACE1 antibody and imaged for BACE1 immunofluorescence (red) and YFP fluorescence (green) by confocal microscopy. Upper row shows lower magnification of several amyloid plaques (stars) each surrounded by an annulus of punctate accumulations of BACE1 and BACE1-YFP. Lower row shows higher magnification image of boxed inset in upper row. Our previous work has identified these BACE1 accumulations as swollen dystrophic axons and presynaptic terminals [24]. Note the extensive co-localization of BACE1 and BACE1-YFP, although their relative levels appear to vary somewhat in different dystrophies. These results demonstrate that BACE1-YFP accumulates around plaques in the same pattern as endogenous BACE1. Blue in the center of the annulus represents the amyloid plaque core, marked with (*). Blue outside of the annulus indicates DAPI-stained nuclei.

Phosphorylated eIF2α increases BACE1 translation by causing ribosome scan-through of three upstream open reading frames (uORFs) present in the 5′ UTR of BACE1 mRNA [46], thus increasing the probabilty of ribosome translation re-initiation at the true BACE1 ORF [22]. A BACE1 transgene with a truncated 5′ UTR lacking the uORFs does not result in increased BACE1 translation in response to energy deprivation stress that induces eIF2α phosphorylation [22]. If Aβ is also a stress that causes eIF2α phosphorylation and increased BACE1 mRNA translation, we reasoned that BACE1-YFP transgenic mice that express a transcript with a truncated BACE1 mRNA 5′ UTR under the control of the CaMKII promoter [52] should not exhibit increased BACE1-YFP levels associated with amyloid. Sequencing demonstrated that only 11 nucleotides of the BACE1 mRNA 5′ UTR and no uORFs are present in the BACE1-YFP transgene 5′ UTR (Fig. 9A). To determine whether or not BACE1-YFP levels are elevated in response to amyloid, we crossed BACE1-YFP transgenics with 5XFAD mice to generate BACE1-YFP; 5XFAD compound transgenic animals, aged them to 6–8 months, and harvested cortices and hemibrains for biochemical and histological analyses. Immunoblot analysis with an anti-BACE1 antibody revealed that BACE1-YFP levels were significantly increased in BACE1-YFP; 5XFAD brain homogenates, as compared to those from age-matched mice that express BACE1-YFP alone (Fig. 9B). Moreover, YFP fluorescence in brain sections from BACE1-YFP; 5XFAD mice demonstrated marked accumulation of BACE1-YFP in swollen dystrophic neurites surrounding amyloid deposits in a pattern identical to endogenous BACE1 immunostaining near plaques in APP transgenic and AD brains (Fig. 5C; [24], [26]). Thus, BACE1-YFP underwent Aβ-dependent elevation in 5XFAD brain, even when expressed from a transgene lacking the 5′ UTR uORFs that are directly responsible for the translational control of BACE1 mRNA by phosphorylated eIF2α. Together with our other genetic evidence, our BACE1-YFP transgenic results strongly support the conclusion that amyloid-associated BACE1 elevation occurs via a mechanism that is independent of eIF2α phosphorylation.

## Discussion

In this report we use three different genetic strategies (GADD34 CA-AAV transduction, eIF2α S51A knockin mutation, BACE1-YFP transgene lacking 5′UTR uORFs) to investigate the potential role of eIF2α phosphorylation in Aβ-dependent BACE1 elevation in primary neuron culture and in the brain of the 5XFAD mouse model of aggressive amyloid pathology. We unequivocally demonstrate that partial or complete reduction of eIF2α phosphorylation does not inhibit the Aβ-associated increase of BACE1 level either in vitro or in vivo. Furthermore, we show that YFP-tagged BACE1, expressed from a transgene with a truncated BACE1 mRNA 5′ UTR lacking uORFs required for p-eIF2α mediated translational control, is elevated and accumulates around plaques in a pattern identical to endogenous BACE1 in 5XFAD brain. Additionally, p-eIF2α reduction did not block Aβ-dependent APP elevation in primary neurons, nor did it decrease Aβ levels or amyloid plaque pathology in 5XFAD brain. Thus, we can conclude that eIF2α phosphorylation does not have a role in Aβ-associated BACE1 and APP elevation, or in amyloid pathology, at least in primary neurons and 5XFAD mice.

Our three distinct genetic strategies produced results that were very consistent with one another, making it highly unlikely that the data were artifacts of the approaches. Taken together, they strongly support the conclusion that amyloid-associated BACE1 elevation does not depend on eIF2α phosphorylation or on any other translational or transcriptional mechanism. Instead, we currently favor a hypothesis of Aβ-dependent BACE1 elevation that is driven at the post-translational level by dysregulation of BACE1 localization and/or turnover. Future investigations will shed light on the elusive mechanism of BACE1 elevation in AD.

It is notable that both BACE1 and APP levels became elevated in primary neurons following exposure to Aβ42 oligomers, suggesting a feed-forward mechanism that establishes a vicious pathogenic cycle of Aβ generation. Here, we did not determine whether APP increased in parallel with BACE1 in the brains of 5XFAD mice. However, we recently demonstrated the accumulation of both BACE1 and APP in dystrophic axons surrounding amyloid plaques in 5XFAD brains [24]. Previous studies suggest that a halo of Aβ oligomers is present around the amyloid plaque [3], implying that neuritic dystrophy in close proximity to the plaque might arise from Aβ oligomer-induced toxicity. Putting these observations together, we speculate that high concentration Aβ42 oligomers emanating from plaques cause peri-plaque neurites to become swollen and dystrophic, leading to the accumulation of BACE1 and APP with subsequent elevated Aβ production.

Our results differ from others suggesting a role for eIF2α phosphorylation in amyloid-associated elevation of BACE1 levels and amyloid pathology. BACE1 and p-eIF2α levels have been reported to increase together under certain genetic and pharmacologic conditions in APP transgenic mice [22], [69]–[71]. However, these observations are essentially correlative and the corresponding studies did not test the effects of direct inhibition of eIF2α phosphorylation on Aβ-induced BACE1 levels, as did our present investigation. For example, in one study 5XFAD mice were generated that lacked the eIF2α kinase GCN4 [70]. 5XFAD; GCN4^−/−^ mice exhibited elevated p-eIF2α, BACE1, and Aβ levels compared to 5XFAD; GCN4^+/+^ littermates. A compensatory increase in the activation of another eIF2α kinase, PERK, was also observed in 5XFAD; GCN4^−/−^ mice, providing an explanation for the p-eIF2α elevation. However, this study did not directly demonstrate that the BACE1 elevation was caused by the increase in p-eIF2α. Indeed, levels of proteins important for the regulation of gene expression such as ATF4 and phosphorylated CREB were also changed in 5XFAD; GCN4^−/−^ mice, suggesting the possibility of alternative pathways of BACE1 elevation independent of p-eIF2α. The other reports also suffer from over-interpretation of correlative results. In support of our results, a recent study using a different APP transgenic mouse reported that genetic ablation of PERK reduced p-eIF2α levels but did not alter BACE1 levels [72]. By direct inhibition of eIF2α phosphorylation, here we definitively tested cause and effect relationships and conclusively show that reducing eIF2α phosphorylation does not decrease amyloid-associated BACE1 elevation in primary neuron cultures or 5XFAD mice.

We note that there are conflicting reports concerning the level of p-eIF2α present in the brains of 5XFAD mice. We previously reported that the p:t eIF2α ratio in 5XFAD mice at 6 months of age was increased modestly (∼20%) compared to non-Tg mice [22]. However, we failed to confirm this finding in the present study and instead showed no significant difference in p:t eIF2α ratio between 6- or 12-month 5XFAD and non-Tg mice using a much larger sample size (n = 9–15 here compared to n = 5 in [22]). Another group has reported that p-eIF2α levels are increased ∼3- and ∼9-fold at 6 and 15–18 months of age, respectively [69]. The reason for these discrepancies in reported p-eIF2α levels in 5XFAD mice is unclear and will require further investigation to resolve. However, it is worth noting that 2–12 month-old 5XFAD mice display robust increases in BACE1 levels despite failing to show consistently elevated p-eIF2α in our hands, again arguing against a definitive role for eIF2α phosphorylation in amyloid-associated BACE1 elevation.

On a technical note, this study demonstrates the effective use of a two-vector AAV system with P0 intracerebroventricular injection for transgene expression in the brain. Previous studies using this injection method have used a single virus, which limits the flexibility of the regulation of the transduced transgene. AAV vectors can package only a limited sized cDNA insert (∼5–6 kb) between the inverted terminal repeats, so our demonstration of the feasibility of AAV co-transduction expands the possibility for more complex regulation and combinations of different transgenes. In our study, AAV co-transduction permitted us to double the amount of exogenous DNA (e.g., for co-expression of the gene of interest with GFP) and perform cell specific expression in neurons coupled with doxycycline regulation (which we did not use in this study).

Our two-vector system requires that individual cells become infected with a sufficient number of each AAV to achieve adequate expression of both vectors. Since we were able to almost completely eliminate eIF2α phosphorylation, it appears that sufficient co-infection levels of GADD34 CA-AAV and CamKII tTA-AAV vectors were attained at the non-toxic titers that we used (see Methods). It is notable that eIF2α phosphorylation was reduced by as much as 90%, suggesting that the majority of p-eIF2α in the brain is found in excitatory forebrain neurons (in which CaMKII tTA-AAV is specifically expressed) rather than in other cell types.

Our recent work here and in another report [25] focused on whether dysregulation of specific cellular mechanisms that control BACE1 levels in neurons is responsible for Aβ-dependent BACE1 elevation. Taken together, our data suggest that while pathways involving eIF2α phosphorylation, Cdk5 activity and Caspase 3 activation, among others have a role in regulating BACE1 levels under normal physiological conditions and stress responses, these mechanisms are not responsible for raising BACE1 levels in an Aβ42 dependent fashion. Clearly, BACE1 translation can be upregulated by different stresses that induce eIF2α phosphorylation, such as energy deprivation [22], and this mechanism is undoubtedly important during certain stress responses. While our work suggests it is highly unlikely that eIF2α phosphorylation is responsible for increased BACE1 levels observed in Aβ42 treated primary neurons or 5XFAD brains, further investigation of this pathway is still relevant as p-eIF2α levels are elevated in the brains of AD patients [22] and could affect AD pathology through mechanisms other than BACE1 elevation.
