# Correction: Genetic Inhibition of Phosphorylation of the Translation Initiation Factor eIF2α Does Not Block Aβ-Dependent Elevation of BACE1 and APP Levels or Reduce Amyloid Pathology in a Mouse Model of Alzheimer’s Disease

**DOI:** 10.1371/journal.pone.0110914

**Published:** 2014-10-14

**Authors:** The PLOS ONE Staff

Figure 1 is incorrect. Please see the corrected Figure 1 here.

**Figure 1:**
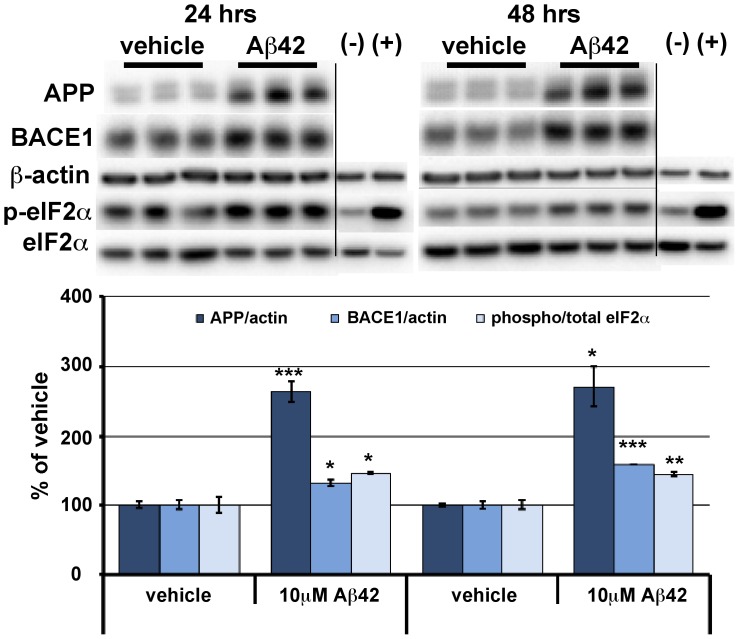
BACE1, APP, and phosphorylated eIF2α increase in response to A β42 oligomer treatment of primary neurons. Mixed cortical primary neurons were isolated from e15.5 mouse embryos, and after 7 days in culture, exposed to 10 µM oligomeric Aβ42 for 24 and 48 hrs. Cells were lysed in RIPA buffer and 10 µg/lane of protein were subjected to immunoblot analysis for APP, BACE1, phosphorylated (p)-eIF2α, total eIF2α, and β-actin as a loading control. (–) and (+) are negative and positive controls for eIF2α phosphorylation (control and UV treated HEK cell lysates, Cell Signaling). APP and BACE1 immunosignal intensities were normalized to those of β-actin. Phosphorylated and total eIF2α immunosignal intensities were measured and phosphorylated:total eIF2α ratio calculated. APP and BACE1 levels and phosphorylated:total eIF2α ratio (all measures displayed as percentage of vehicle control) are all significantly elevated by Aβ42 oligomer treatment at both time points. Bars represent SEM, n  =  3 samples per condition, asterisks (*) indicate significant changes compared to respective vehicle, p<0.05*, p<0.01**, p<0.001***.

Figure 9 is incorrect. Please see the corrected Figure 9 here.

**Figure 9:**
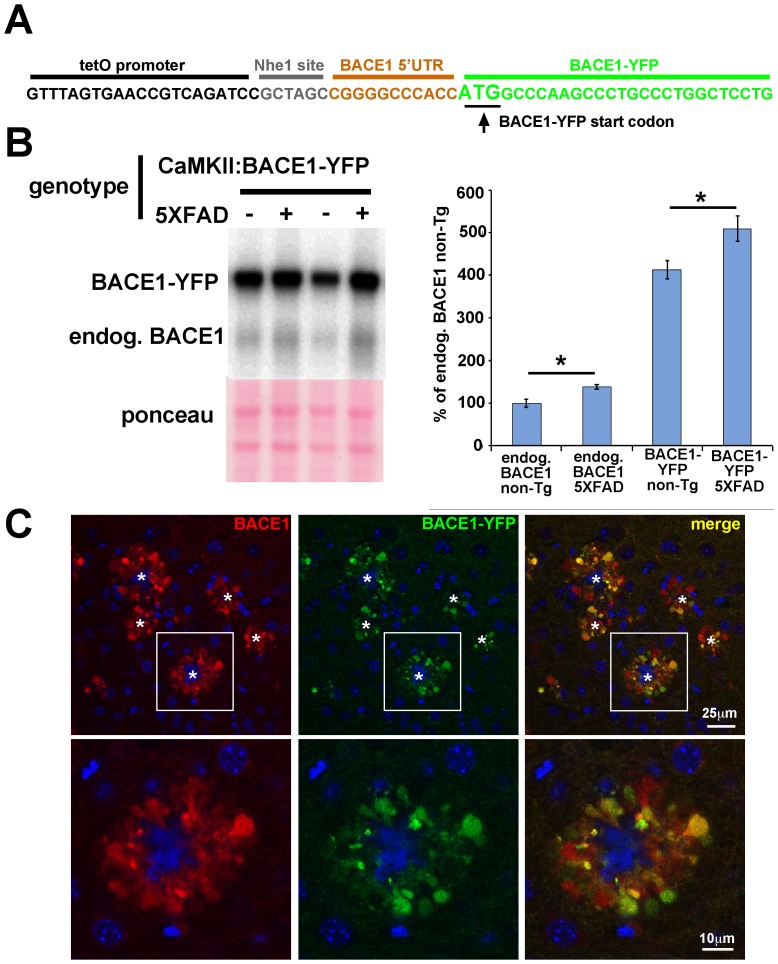
BACE1-YFP expressed from a transgene with a truncated BACE1 mRNA 5′ UTR is also elevated and accumulates around amyloid plaques in 5XFAD brain. (A) 5′ UTR of BACE1-YFP transgene. The BACE1-YFP coding region (green) was subcloned into the tetO promoter vector PMM400 (black) via an NheI site (gray) [52], leaving a severely truncated BACE1 mRNA 5′ UTR (orange) consisting of only eleven nucleotides that lack the uORFs required for de-repression of translation by phosphorylated eIF2α. (B) 5XFAD mice were crossed with BACE1-YFP transgenic mice to generate 5XFAD (+) and non-Tg (–) offspring that also expressed the BACE1-YFP transgene. 5XFAD and non-Tg offspring that lacked the BACE1-YFP transgene were also generated. At 6–8 months of age, cortices of 5XFAD; BACE1-YFP, non-Tg; BACE1-YFP, 5XFAD, and non-Tg littermates (n  =  5 for each group) were harvested, homogenized, and 20 µg/lane of homogenates were subjected to immunoblot analysis for BACE1 using the 3D5 anti-BACE1 antibody. The immunoblot was stained with ponceau S as a protein loading control. Representative BACE1-YFP immunoblot signals are shown. BACE1-YFP runs at ∼90 kDa on SDS-PAGE, compared to ∼65 kDa for endogenous (endog.) BACE1. For quantification, BACE1 and BACE1-YFP immunosignal intensities were normalized to ponceau S staining intensity for a given lane. The means of each group were calculated and means displayed as percentage of the mean BACE1 level in non-Tg control. The BACE1-YFP transgene is expressed at levels that are ∼4-fold higher than that of endogenous BACE1. As expected, endogenous BACE1 level is significantly elevated in 5XFAD brain compared to non-Tg brain. Most importantly, BACE1-YFP levels also exhibit a significant amyloid-associated elevation with the 5XFAD genotype compared to the non-Tg genotype, despite the complete absence of uORFs necessary for regulation by eIF2α phosphorylation. Bars represent SEM, asterisks (*) indicate significant changes compared to respective non-Tg control, p<0.05*. (C) Sagittal section of representative 5XFAD; BACE1-YFP cortex stained with anti-BACE1 antibody and imaged for BACE1 immunofluorescence (red) and YFP fluorescence (green) by confocal microscopy. Upper row shows lower magnification of several amyloid plaques (stars) each surrounded by an annulus of punctate accumulations of BACE1 and BACE1-YFP. Lower row shows higher magnification image of boxed inset in upper row. Our previous work has identified these BACE1 accumulations as swollen dystrophic axons and presynaptic terminals [24]. Note the extensive co-localization of BACE1 and BACE1-YFP, although their relative levels appear to vary somewhat in different dystrophies. These results demonstrate that BACE1-YFP accumulates around plaques in the same pattern as endogenous BACE1. Blue in the center of the annulus represents the amyloid plaque core, marked with (*). Blue outside of the annulus indicates DAPI-stained nuclei.
